# Transcryptomic Analysis of Human Brain-Microvascular Endothelial Response to -Pericytes: Cell Orientation Defines Barrier Function

**DOI:** 10.3390/cells10040963

**Published:** 2021-04-20

**Authors:** Lisa Kurmann, Michal Okoniewski, Omolara O. Ogunshola, Brigitte Leeners, Bruno Imthurn, Raghvendra K. Dubey

**Affiliations:** 1 Department of Reproductive Endocrinology, University Hospital Zurich, 8952 Schlieren, Switzerland; lisa.kurmann@usz.ch (L.K.); brigitte.leeners@usz.ch (B.L.); bruno.imthurn@uzh.ch (B.I.); 2 ID Scientific IT Services, ETH Zurich, 8092 Zurich, Switzerland; michal.okoniewski@id.ethz.ch; 3 Zurich Center Integrative Physiology (ZIHP), Vetsuisse Faculty, Institute of Veterinary Physiology, University of Zurich, 8057 Zurich, Switzerland; larao@access.uzh.ch; 4 Department of Pharmacology & Chemical Biology, University of Pittsburgh, Pittsburgh, PA 15219, USA

**Keywords:** pericyte, hCMEC/D3, co-culture, blood–brain barrier, orientation, transcriptome, conditioned media, micro array

## Abstract

Pericytes facilitate blood–brain barrier (BBB) integrity; however, the mechanisms involved remain unclear. Hence, using co-cultures of human cerebral microvascular endothelial cells (ECs) and vascular pericytes (PCs) in different spatial arrangements, as well as PC conditioned media, we investigated the impact of PC-EC orientation and PC-derived soluble factors on EC barrier function. We provide the first evidence that barrier-inducing properties of PCs require basolateral contact with ECs. Gene expression analysis (GEA) in ECs co-cultured with PCs versus ECs alone showed significant upregulation of 38 genes and downregulation of 122 genes. Pathway enrichment analysis of modulated genes showed significant regulation of several pathways, including transforming growth factor-β and interleukin-1 regulated extracellular matrix, interferon and interleukin signaling, immune system signaling, receptor of advanced glycation end products (RAGE), and cytokine–cytokine receptor interaction. Transcriptomic analysis showed a reduction in molecules such as pro-inflammatory cytokines and chemokines, which are known to be induced during BBB disruption. Moreover, cytokine proteome array confirmed the downregulation of key pro-inflammatory cytokines and chemokines on the protein level. Other molecules which influence BBB and were favorably modulated upon EC-PC co-culture include IL-18 binding protein, kallikrein-3, CSF2 CSF3, CXCL10, CXCL11 (downregulated) and IL-1-R4; HGF, PDGF-AB/BB, PECAM, SERPIN E1 (upregulated). In conclusion, we provide the first evidence that (1) basolateral contact between ECs and PCs is essential for EC barrier function and integrity; (2) in ECs co-cultured with PCs, the profile of BBB disrupting pro-inflammatory molecules and cytokines/chemokines is downregulated; (3) PCs significantly modulate EC mechanisms known to improve barrier function, including TGF-β regulated ECM pathway, anti-inflammatory cytokines, growth factors and matrix proteins. This human PC-EC co-culture may serve as a viable in vitro model for investigating BBB function and drug transport.

## 1. Introduction

The neurovascular unit (NVU) constitutes a highly evolved network of many different cell types that together form a selectively permeable boundary between the systemic circulation and the interstitial fluid of the brain, the so-called blood–brain barrier (BBB). In addition to a single layer of endothelial cells (ECs), also astrocytes, pericytes (PCs), microglia and neurons hold critical functions in maintaining the integrity of the vessel wall [1,2]. PCs have long been an overlooked player in the field of BBB research. More recently, however, these mural cells have gained increasing attention as several studies have revealed the importance of PCs with regard to a properly functioning endothelial barrier. In vivo studies using viable pericyte-deficient mouse models provide evidence that PCs are critical for BBB formation and PC loss results in severe vessel leakage [1,2]. Additionally, PC degeneration is associated with several central nervous system (CNS) disorders such as Alzheimer’s disease, stroke or amyotrophic lateral sclerosis (ALS) [3]. Apart from PC depletion, disturbed signaling between ECs and PCs contributes to barrier leakage and disease states. Communication between the two cell types is known to occur via paracrine signaling involving a plethora of soluble factors, but also direct cell-to-cell contacts in form of so-called peg-socket interactions, gap junctions and adhesion plaques play a significant role [4]. 

Transport across the endothelial barrier can be partitioned into two major pathways: paracellular and transcellular [5]. The paracellular permeability is restricted to small, water-soluble molecules with a Stoke’s radius of less than 3 to 6 nm and is regulated by a complex interplay between different junctional proteins and the actin cytoskeleton of cells [5,6]. Transcellular routes, which include the steps of endocytosis, transport across the cell lumen and exocytosis, are mainly responsible for macromolecular transport. The uptake of molecules can be carrier- or receptor-mediated or non-specific via adsorption-mediated endocytosis or pinocytosis [5,7]. Understanding the regulatory mechanisms of transport pathways across the endothelial barrier is of great importance, particularly in the field of drug delivery into the central CNS. Several promising therapeutics developed for CNS diseases have failed in clinical trials due to the lack of an appropriate delivery system across the BBB [7,8].

Due to the high complexity of in vivo data analysis, researchers make use of different in vitro models for the BBB that include a combination of two or more cell types of the NVU [9]. Although these models represent a vastly simplified picture of the in vivo situation, they are of great importance when it comes to understand the contribution of a single cell type to the function of the BBB [9]. Using such simplified 2D models, many studies have improved our basic understanding of how PCs affect the function of the endothelial barrier and, importantly, the mechanisms behind it [10,11,12]. Observed variations in published data may in part be explained by the various possibilities of assembling EC-PC co-culture models in different constellations. 

To test our hypothesis that PCs interact with ECs to improve barrier function, we first investigated the impact of different spatial arrangements of ECs and PCs on the function of the endothelial barrier. Specifically, we seeded the PCs on the opposite side of the endothelial cell layer on the basolateral side of the transwell insert (indirect) or together with the ECs on the apical side in direct contact. Moreover, a non-contact model with PCs plated on the well surface was also included. Additionally, we added pericyte-conditioned media (PCM) to inserts culturing endothelial monolayers in order to evaluate the effect of soluble factors secreted by PCs. Using different approaches for measuring barrier function, we assessed whether PCs modulate para- and/or transcellular transport pathways in ECs. Furthermore, using transcriptomic gene expression analysis and proteome arrays, we explored potential signaling pathways and modulatory molecules by which PCs improve EC barrier function.

## 2. Materials and Methods

### 2.1. Cell Culture

hBVPs: Human Brain Vascular Pericytes (HBVPs, ScienCell, CA, USA) between 4–10th passage were cultured in Poly-L-Lysine- (PLL-) coated flasks (2 µg/cm^2^) under standard tissue culture conditions (37 °C, 5% CO_2_) in growing media consisting of DMEM/F12 supplemented with antibiotic-antimycotic (AA; 100 μg/mL streptomycin, 100 μg/mL penicillin and 0.025 μg/mL amphotericin B), Glutamax (1×) and 10% FBS. Media was changed every two or three days until sub-confluency.

hCMEC/D3: The human Cerebral Microvascular Endothelial Cell line (hCMEC/D3) [13] was kindly provided by Dr. Pierre-Olivier Couraud (Institute COCHIN, Paris, France). Cells between 34th and 39th passage were cultured on rat-tail-collagen-coated (250 μg/mL in 80% EtOH) flasks under standard tissue culture conditions (37 °C, 5% CO_2_) in complete growing media (EC basal media (EndoGRO Basal Medium supplemented with 0.2% EndoGRO-LS Supplement, 5 ng/mL rh EGF, 4 mM L-Glutamine, 0.75 U/mL Heparin Sulfate, 50 μg/mL Ascorbic Acid, 1 ng/mL bFGF, antibiotic-antimycotic (100 μg/mL streptomycin, 100 μg/mL penicillin and 0.025 μg/mL amphotericin B))) supplemented with 5% FBS. Media was changed every two or three days, and cells were passaged after confluency was reached. 

hASMC: Human Coronary Artery Smooth Muscle Cells (LONZA, Walkersville, MD, USA) between 6–9th passage were cultured in non-coated flasks under standard tissue culture conditions (37 °C, 5% CO_2_) in growing media consisting of M231 culture medium supplemented with antibiotic-antimycotic (AA; 100 μg/mL streptomycin, 100 μg/mL penicillin and 0.025 μg/mL amphotericin B) and SMGS (5% *v/v* FCS, 2 ng/mL human basic Fibroblast Growth Factor, 0.5 ng/mL human Epidermal Growth Factor, 5 ng/mL Heparin, 5 μg/mL insulin and 0.2 μg/mL BSA). Media was changed every two or three days until sub-confluency. 

### 2.2. Barrier Function Studies

Determination of endothelial cell barrier function was achieved by three different methods: Trans-endothelial electric resistance measurements (TEER), macromolecular tracer assays and electric cell substrate impedance measurements.

### 2.3. Transendothelial Electric Resistance (TEER)

For TEER measurements in real-time, a cellZcope instrument (nanoAnalytics GmbH, Münster, DE) was used. Permeable transparent PET membrane inserts of 0.4 µm pore size and 24-well format (Corning Incorporated, NY, USA, Falcon 353095) were coated with poly-L-Lysine on the basolateral side and with rat tail collagen (250 μg/mL in 80% EtOH) on the apical side for 1h at 37 °C and washed twice with sterile H_2_O, before they were incubated for another hour in PC growing media (5% FBS). For the co-culture models, hBVPs were seeded either on the basolateral or on the apical side of the insert at a density of 25,000 cells/cm^2^. After adherence of the PCs was achieved (3 h), hCMEC/D3 were seeded in the apical chamber at a cell density of 100,000 cells/cm^2^ in 150 µL EndoGro growing media. This set-up allows the two cell types to form direct contacts through the pores of the membrane [14]. Media in the basolateral chamber consisted of PC growing media (5% FBS). After 3 days, media was changed to 2% FBS and hydrocortisone (1 μg/mL) was added. For measurements, inserts were added to the cellZcope instrument (lower chamber: 1 mL media, upper chamber: 0.5 mL media). Establishment of a proper barrier function was monitored by recording resistance values of the inserts every hour, and treatments, when necessary, were only applied once the cells reached a stable baseline. All measurements were normalized to the values of a coated insert without any cells and results are depicted in% of control or in absolute TEER values in ohm*cm^2^.

### 2.4. Macromolecular Tracer Assay 

For the macromolecular tracer assay, cells were seeded into the inserts as described above. Treatments, when necessary, were only applied after cells reached stable TEER baseline as was determined by measurements with the cellZcope instrument. FITC-labeled dextran molecules (1 mg/mL in media) of two different sizes (4 and 40 kDa) were added to the upper chamber of the inserts in 100 μL/insert, with 500 μL of media in the basolateral chamber. Samples (100 μL) were collected from the basolateral chamber after 40 min and added to a fluoro-block 96-wellplate. Fluorescent intensity was determined by using a Tecan Spectrofluorometer reader (Tecan, Salzburg, AU) (excitation at 490 nm and emission at 520 nm). Relative fluorescent intensity was determined by normalizing to the control sample.

### 2.5. Electric Cell Substrate Impedance Sensing (ECIS)

Impedance measurements were performed with the xCELLigence RTCA system (Omni Life Sciences, Basel, CH). Wells of 16-well E-plates were coated with 40 μL rat-tail-collagen (250 μg/mL in 80% EtOH) and/or PLL (2 µg/cm^2^) for 1 h at 37 °C, washed 2× with sterile ddH_2_O and subsequently incubated with 50 μL of EC growing media for at least 1 h in the tissue culture incubator. The baseline was measured in each well according to manufacturer’s protocol, before hCMEC/D3 cells were seeded at a density of 100,000 cells/cm^2^ in 100 μL growing media. Impedance was recorded in real time every hour or as specified and was converted to the dimensionless parameter cell index (arbitrary unit) by the xCELLigence RTCA software. Noted values on the *y*-axis were labeled as cell index (or normalized cell index, if normalized to the timepoint before treatment).

### 2.6. Conditioned Media (CM) 

Pericyte (PC-) and smooth muscle cell (SMC-) CM.

HBVPs and hASMCs were cultured in 75 cm^2^- (or 25 cm^2^-) culturing flasks in growing media until confluency was reached. After 2 washing steps with HBSS (+Mg^2+^ +Ca^2+^), 10 mL (or 3.3 mL) serum-free DMEM/F12 supplemented with Glutamax (1×) was added for 24 h. CM was collected and centrifuged at 1000× *g* for 10 min at 4 °C. The supernatant was carefully collected, aliquoted and frozen immediately at ×80 °C until further use. Control consisted of non-conditioned media, which was not added to cells, but prepared at the same time as the CM and otherwise treated identically. For CM-treatments, the collected CM was supplemented with Glutamax (1×), antibiotic-antimycotic (AA; 100 μg/mL streptomycin, 100 μg/mL penicillin and 0.025 μg/mL amphotericin B) and 5% or 2% FBS, depending on the experiment.

#### Co-Culture-Conditioned Media (Co-CM)

HBVPs were cultured on the basolateral side of transwell inserts with or without endothelial cells on the apical side for 5 days, before inserts were removed (same media conditions as used in normal co-culture experiments on inserts). New inserts containing established endothelial monolayers were then added to the wells containing conditioned media.

### 2.7. Cell Count

hCMEC/D3 cells were plated in 48-wellplates and allowed to grow to confluence before treatment was added for the specified period of time. Thereafter, cells were washed 2× with HBSS (-Mg^2+^ -Ca^2+^), trypsinized with 0.5% trypsin and counted with a Coulter Counter (Coulter Electronics, Luton, UK). Relative cell number was assessed by normalizing to the control treated samples.

### 2.8. Crystal Violet Staining (CVS)

hCMEC/D3 cells were plated in 48-wellplates and allowed to grow to confluence. After treatment, paraformaldehyde (PFA, 4%) was added to the media (1:1) for 2 min, before media was removed and fresh PFA (4%) was added for 15 min at RT without shaking. Subsequently, cells were washed once with HBSS (+Mg^2+^ +Ca^2+^), and twice with ddH_2_O for 3 min each wash. Crystal violet staining (CVS) solution (0.5% aqueous solution in 25% Methanol) was added for 10 min at RT while shaking slightly. Cells were washed extensively with ddH_2_O until no coloring of the water was visible anymore. Solubilization of the cells was achieved by adding 1% SDS aqueous solution while shaking at RT for about 5 to 10 min. The solution was transferred to a 96-wellplate for reading the absorbance at 595 nm using a Tecan Spectrofluorometer reader (Tecan, Salzburg, AU).

### 2.9. Microarray Analysis

For the microarray samples, ECs were seeded alone or with PCs on the opposite side of permeable PET membrane inserts of 0.4 µm pore size and 6-well format (Corning Incorporated, NY, USA, Costar 3450). After 7 days in culture (2% steroid-free FCS (charcoal-stripped) in presence of hydrocortisone), cells were trypsinized, centrifuged and lysed in 300 μL RNA lysis buffer (Zymo Research, CA, USA). Samples were frozen at −80 °C until further processing. Total RNA was then extracted by using the Quick-RNA MiniPrep Kit (ZymoResearch, CA, USA, R1055) according to the manufacture’s protocol. RNA integrity was checked by calculating the ratio of absorbance at 260 nm and 280 nm/230 nm, respectively. The samples were frozen at −80 °C for microarray analysis using Affymetrix Clariom S Assay, human (Applied Biosystems by Thermo Fisher Scientific Inc, OK, USA, 902927) as previously described [15]. For transcriptome analysis, fragmented biotin-labeled ds cDNA was hybridized to Clariom™ S arrays (Clariom™ S arrays, human). After staining, arrays were scanned with Affymetrix Gene-Chip Scanner-3000-7G while quality control analysis was performed using GeneChip Command Console Software (GCC) v5.0. Transcriptome analysis was done at the transcriptomics core facility at the Center for Molecular Medicine Cologne (CMMC). Differentially regulated genes were determined with the Transcriptome Analysis Console (TAC, Applied Biosystems by Thermo Fisher Scientific Inc, OK, USA) after uploading the CEL files, based on a foldchange cut-off of ± 1.5 (Log2 FC ± 0.59) and FDR P-value of 0.05. Pathway analysis was performed using NCATS BioPlanet on the Enrichr website, which includes more than 1600 human pathways from publicly available sources [16]. The microarray data are deposited in the public Gene Expression Omnibus (GEO) database under the accession no. GSE168514 (access date 19th April 2021).

### 2.10. Cytokine Proteome Array

For analysis of cytokine expression in co- and mono-cultured ECs, the Proteome Profiler Human XL Cytokine Array Kit (R&D Systems, MN, USA, ARY022B) was used. Cells were grown exactly as described in the microarray section above. After trypsinization and centrifugation, the pellet was lysed in lysis buffer 17 (R&D Systems, MN, USA) supplemented with aprotinin, leupeptin and pepstatin (10 µg/mL each), and after processing according to the user’s manual, they were frozen at −80 °C until further use. Incubation of the ready-to-use membranes was done o/n with equal amounts of samples (175 µg in 1.5 mL), as was determined by BCA analysis. The following detection of the proteins was performed exactly as described in the user’s manual and for the exposure of the membranes Hyperfilm ECL (Amersham, CH) were used in a CAWOMAT 2000 IR film developer (Wiroma AG, Niederscherli, CH). Experiment was performed two times with independently prepared samples. Signal density of each spot was determined with the imageJ software after background subtraction and values represented in the graphs denote mean values of each protein from the two experiments. 

### 2.11. Western Blot Analysis

For Western blot analysis, cells were grown exactly as described in the microarray section above. After trypsinization and centrifugation, the pellet was lysed in lysis buffer (containing 20 mM Tris pH 7.5, 1% Triton X- 100, 150 mM NaCl, 1 mM EGTA, 1 mM EDTA, 2.5 mM sodium phosphate, 1 mM β-glycerophosphate, 1 mM sodium vanadate, 0.5 PMSF and 0.2% SDS). Concentration of each sample was determined with the Pierce bicinchoninic acid (BCA) Assay Kit according to the manufacturer’s protocol. Equivalent amounts (10 μg) of protein from whole-cell lysates were separated on 10% SDS-polyacrylamide gels. After transfer to a nitrocellulose membrane by the method of wet electroblotting, the membrane was blocked with 5% milk at RT for 1h. Incubation with the primary antibody was performed o/n at 4 °C. After washing, the membrane was incubated with the secondary antibody for 1h at RT and washed again. For detection of proteins with IR Dyes, the Odyssey LI-COR system (LI-COR, Nebraska, USA) was used. For peroxidase-conjugated secondary antibodies, chemiluminescent substrates (Pierce, Rockford, USA) were added according to manufacturer’s instruction. Peroxidase activity was detected by exposing the membranes to XOMAT LS films, which were developed with the CAWOMAT 2000 IR film developer (WIROMA AG, Niederscherli, CH).

### 2.12. Statistical Analysis

Experiments were performed at least 3 times, and data are represented as mean ± SD unless stated otherwise. Statistical evaluation was performed by using R. If ANOVA assumptions were met parametric testing was performed with one-way ANOVA and subsequent Tukey’s HSD multiple pairwise comparisons. If either one of the ANOVA assumptions were not met, non-parametric testing was performed with Kruskal–Wallis rank sum test and subsequent pairwise Wilcoxon-test with Benjamini–Hochberg corrections for multiple comparisons. Microarray data analysis was performed with the Transcriptome Analysis Console (TAC, Applied Biosystems), which uses generalized linear models (Limma-based, eBayes). The generation of probe set intensity values was performed by ‘Signal Space Transformation’ (SST)-RMA normalization method and background correction.

## 3. Results

### 3.1. Preliminary Experiments

In the following, the barrier function of endothelial cells (ECs) is measured by three different methods: trans-endothelial electric resistance measurements (TEER), macromolecular tracer assays and electric cell substrate impedance sensing (ECIS) with the xCELLigence system. While the first two systems are well established and most-commonly used for in vitro barrier function measurements, the used ECIS system (xCELLigence RTCA) is usually used for monitoring cell growth, adherence or motility. However, several studies have also applied the same system for measuring barrier function [17,18,19,20]. In the Supplementary Materials, we provide measurements that demonstrate the usability of the system and show that for example hydrocortisone, a well-known barrier-inducer [21], increases barrier function as measured with this system. To demonstrate barrier disruption, we used hypoxic conditions as well as the inflammatory mediator thrombin to show a decrease in the measured barrier function (Supplementary Figure S1). 

### 3.2. Effect of Different Co-Culture Arrangements on Barrier Function

**Figure 1:**
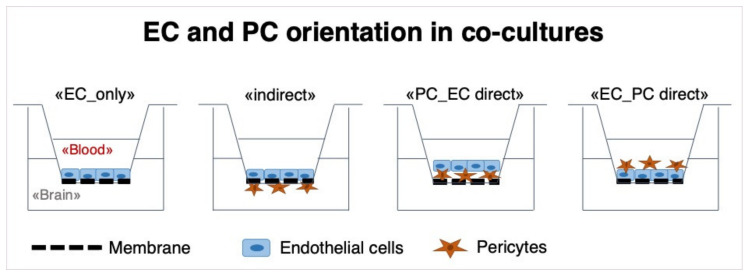
Schematic illustration of the experimental design of BBB models that were used in this study. “EC_only“: endothelial cells (ECs) are plated on the apical side of a 24-well-plate insert with a pore-size of 0.4 μm. “indirect“: Pericytes (PCs) are plated on the basolateral side of the insert, and in a second step (after 3 h), ECs are plated on the apical side, allowing contact of the two cell types through the membrane. “PC_EC direct“: PCs are plated on the apical side of inserts first, and after 3 h, ECs are plated on top of PCs. “EC_PC direct“: ECs are plated first, and after 3 h, PCs are seeded on top of them.

In a first step, we examined the effect of different spatial pericyte-endothelial cell (PC-EC) arrangements on barrier function. ECs were always plated on the apical side of the transwell inserts, while PCs were plated either on the basolateral side (“indirect”) or on the apical side in direct contact with ECs (direct). For the direct model, cells were plated in two different constellations: PCs plated first (PC_EC direct) or ECs plated first (EC_PC direct) (Figure 1).

In order to assess the impact of the different models on the barrier function, TEER was measured by means of a cellZcope instrument. In addition, the permeability of the barrier towards FITC-labelled dextran molecules in different sizes (4 kDa and 40 kDa) was assessed. 

As the ratio of PCs to ECs can vary greatly according to different locations and sources even within the brain [3,4,22], barrier function was assessed using different ratios of PCs to ECs (1:1, 1:2 and 1:4), all of which increased the barrier function to a similar extent (Supplementary Figure S2). In subsequent experiments, a ratio of 1:4 (PCs:ECs) was used.

**Figure 2:**
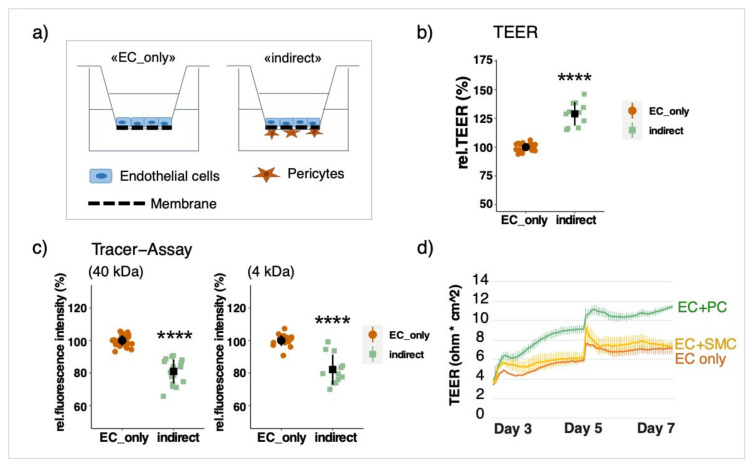
Pericytes (PCs) increase barrier function of endothelial cells (ECs). Schematic representation of the indirect co-culture model (**a**) ECs were cultured on transwell inserts alone (EC_only) or with PCs (indirect) (**b**,**c**) and SMCs (**d**) on the basolateral side of the insert (indirect). Cells were cultured for 7 days before barrier function was assessed. Relative trans-endothelial electric resistance (TEER) measurements were performed with a CellZcope instrument (**b**)**.** Relative fluorescence intensity measured by macromolecular tracer assay with FITC-labeled dextran molecules of 40 and 4 kDa (**c**). Representative real-time TEER measurement of ECs alone and in co-culture with PCs or SMCs (**d**). Experiments were performed at least three times in triplicates and data represent mean ± sd. **** *p* < 0.0001, compared to “EC_only”.

Growing the cells in the indirect co-culture model resulted in a significant upregulation of resistance measurements and a simultaneous decrease in permeability towards FITC-dextran, if compared to an endothelial monolayer (Figure 2b,c). This clearly indicates that the barrier function is enhanced in presence of PCs. In order to assess whether the observed improvement is not only due to the additional cell layer, co-culture experiments with smooth muscle cells (SMCs) instead of PCs were also performed. SMCs had no effect on barrier function as can be derived from Figure 2d. Additionally, the measured TEER value of co-cultured cells was higher than cumulative values for EC and PC monocultures (Supplementary Figure S3).

**Figure 3:**
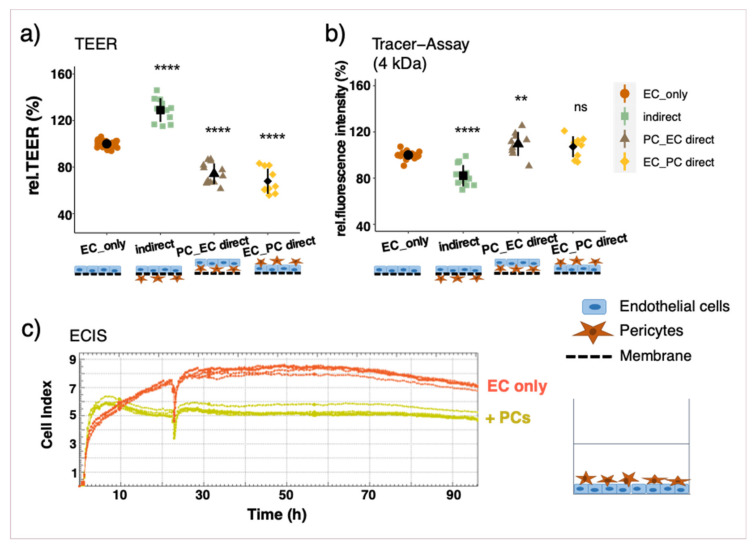
Measurements of endothelial barrier function with and without pericytes (PCs) in different constellations. Endothelial cells (ECs) were cultured on transwell inserts alone (EC_only), with PCs on the opposite side of the insert (indirect) or on the same side (PC_EC direct: PCs seeded first; and EC_PC direct: ECs seeded first). Cells were cultured for 7 days before barrier function was assessed. Relative trans-endothelial electric resistance (TEER) measurements with a CellZcope instrument (**a**). Relative fluorescence intensity measured by macromolecular tracer assay with FITC-dextran 4 kDa (**b**); results for 40 kDa FITC-dextran are comparable (data not shown). Representative ECIS measurements in real-time with the xCELLigence System: ECs were seeded into eplates 3 h before PCs were added on top (**c**). Experiments have been performed at least three times in triplicates and data represent mean ± sd. ** *p* < 0.01, **** *p* < 0.0001, compared to “EC_only”.

If PCs were cultured on the same side of the insert together with ECs, TEER measurements were lower than in EC monolayers, which was accompanied by a higher permeability towards tracer molecules. This observation was irrespective of seeding order (Figure 3a,b) and also applied when PCs were seeded on top of an established EC monolayer (Supplementary Figure S4a). The results of the direct co-cultures were further verified by means of ECIS measurements with the xCELLigence system in real-time. Impedance measurements were significantly lower if PCs were co-cultured together with ECs in any seeding order with direct contact (Figure 2c, Supplementary Figure S4b).

### 3.3. Effect of Pericyte-Conditioned Media on Barrier Function

**Figure 4:**
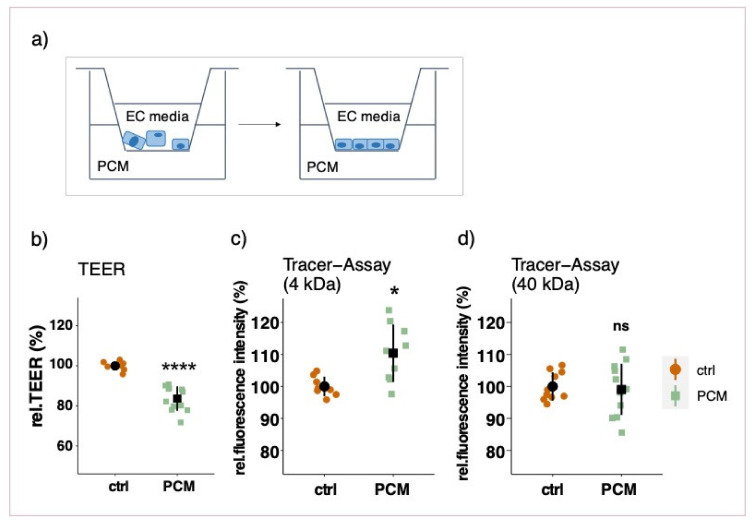
Measurements of endothelial barrier function after prolonged basolateral PCM treatment. Endothelial cells (ECs) were plated on transwell inserts with EC growing media in the apical chamber and PCM in the basolateral chamber (**a**). Relative TEER measurements (**b**) and macromolecular tracer assays with FITC-labeled dextran of 4 and 40 kDa (**c**,**d**) were performed after 7 days in culture. Measurements were performed at least three times in triplicates and data represent mean ± sd. ns *p* > 0.05, * *p* < 0.05, **** *p* < 0.0001.

To assess a possible involvement of soluble factors secreted by PCs, the barrier function of EC monolayers was measured after treatment with PC-conditioned media (PCM) in different set-ups. Treating an established endothelial monolayer with PCM in the basolateral chamber for three days did not have a significant impact on barrier function (Supplementary Figure S5) as measured by TEER and tracer assays. To test whether a prolonged treatment with PCM is necessary for barrier improvement, ECs were treated with PCM as soon as seeded into the inserts. Contrary to our expectations, the barrier function was significantly compromised as shown by a reduction in TEER and by an increased permeability to 4 kDa FITC-dextran (Figure 4b,c). The permeability to bigger FITC-dextran molecules, however, was not affected (40 kDa) (Figure 4d). A similar effect was observed when PCs were plated on the bottom of the well-plate with no direct contact to ECs (non-contact model) as is shown by a decrease in TEER (Supplementary Figure S6). This non-contact model reflects a similar condition as the prolonged PCM treatment. Furthermore, the effect of conditioned media from co-cultured and mono-cultured PCs was compared with respect to barrier function. By treating EC monolayers with PCM from co-cultured and mono-cultured PCs on the basolateral side for 3 days, no differential effect on barrier function was observed (Supplementary Figure S7).

**Figure 5:**
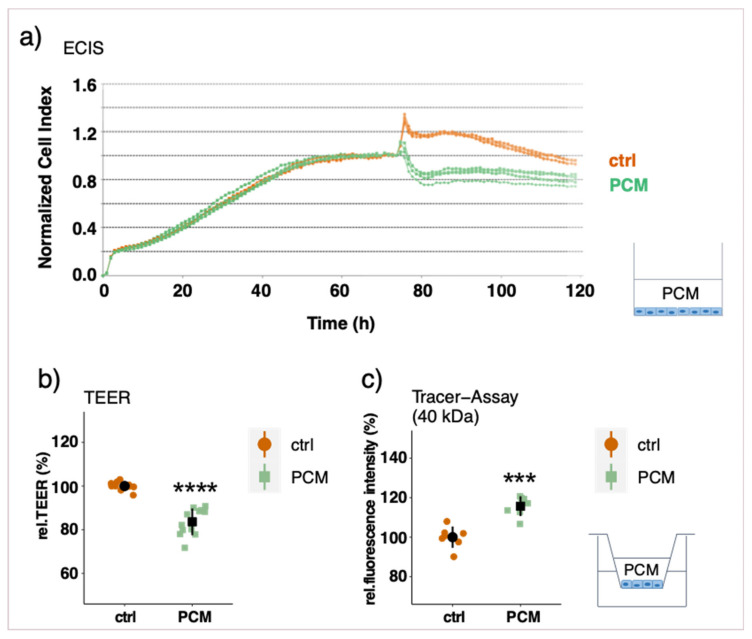
Measurements of endothelial barrier function after apical PCM treatment of an EC monolayer. ECs were cultured in eplates (**a**) or on transwell inserts (**b**,**c**). After a stable barrier function was established (6–7 days), PCM was added to the apical side for 24 h. Representative real-time ECIS measurements with the xCELLigence system (**a**), relative trans-endothelial electric resistance (TEER) measurements with a CellZcope instrument (**b**) and macromolecular tracer assays with FITC-labeled dextran of 40 kDa (representative also for 4 kDa) (**c**). Experiments were performed at least three times in triplicates and data represent mean ± sd. *** *p* < 0.001, **** *p* < 0.0001.

PCM treatment on the apical side of an established EC monolayer led to a significant decrease after only 6 h of treatment as shown by real-time impedance measurement as well as TEER measurements (Figure 5a,b). These findings are further supported by an increase in permeability towards FITC-dextran molecules of 4 and 40 kDa (Figure 5c, 40 kDa as representative for both FITC-dextran sizes). When ECs were apically treated with conditioned media from SMCs, the same effect was observed (Supplementary Figure S8). This suggests a general rather than a pericyte-specific influence, which is more likely related to EC’s apico-basal polarity. 

For the purpose of investigating whether the effect of apical PCM treatment is a result of reduced cell viability, crystal violet staining (CVS) as well as cell counting experiments were performed. The results of both assays clearly showed that PCM treatment did not decrease cell viability after 6 or 48 h of treatment (Supplementary Figure S9). 

### 3.4. Gene Expression Array

**Figure 6:**
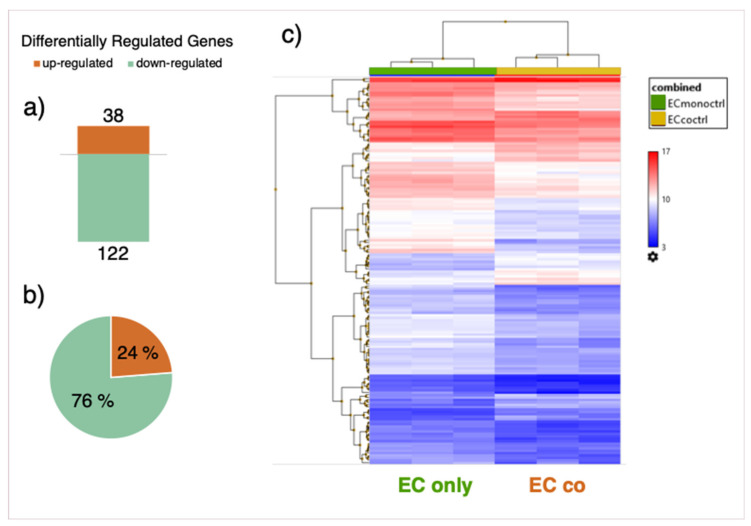
Differentially regulated genes (DRGs). Number of DRGs in co-cultured vs. mono-cultured endothelial cells (ECs). A total of 38 genes were upregulated, while 122 genes were downregulated (**a**). Pie chart representation of the same data as in (**a**) denoting up-and downregulated genes in% of total number of DRGs (**b**). Heat map representation of DRGs (**c**). Comparison of gene expression data in co-cultures vs. mono-cultured ECs was performed by using Transcriptome Analysis Console (TAC, Applied Biosystems). For the analysis, a fold change (FC) cut-off of 1.5 (log2FC ± 0.59) and an FDR p-value of 0.05 were applied.

In order to deepen our knowledge about genes which play a role in improving barrier function in co-cultured cells, transcriptomic analysis of ECs cultured alone and in the indirect co-culture model with PCs was performed by means of a microarray. Differentially regulated genes (DRGs) were determined by comparison of gene expression in co- vs. mono-cultured cells (Supplementary Figure S10). Quality control by means of principle component analysis (PCA) of the different samples shows a clear-cut difference between samples from EC mono- and co-cultures (Supplementary Figure S11). A total of 160 DRGs were detected, with 122 genes down- and 38 genes upregulated (Figure 6a,b). A list of the top ten genes up- and downregulated in co-cultured ECs is provided in the Supplementary Materials (Supplementary Tables S1 and S2). 

Pathway enrichment analysis on DRGs revealed a major impact of co-culture on extra cellular matrix (ECM) regulation as well as on changes of inflammatory pathways (Table 1). 

Possible influential DRGs with regard to barrier function based on literature search are shown in Table 2. The list includes several pro-inflammatory cytokines like IL-1-α, IL-1-β, IL-32 and chemokine ligands-5, -6, -8 and -10 as well as VEGF-C, which are all transcriptionally downregulated in co-cultured cells. Other soluble factors regulated in co-cultured ECs comprise TGF-β2, the NOTCH ligand jagged-1 as well as BMP-4 and BMP-6. When looking at known junctional proteins, we found occludin and claudin-1 to be downregulated significantly on the transcriptional level upon co-culture. Furthermore, cell membrane receptors for leptin and lysophosphatidic acid are up- and downregulated, respectively. Additionally, co-cultured cells differentially express many ECM constituents, several of which are TGF-β -regulated, such as latent TGF-β binding protein 1, serpins, biglycan and tissue plasminogen activator.

**Figure 7:**
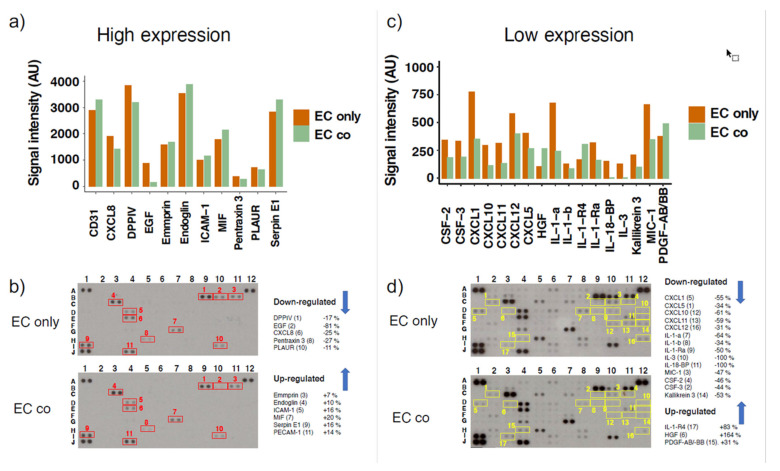
Differential expression of several cytokines in mono- and co-cultured endothelial cells (ECs). Proteome analysis has been performed using the Proteome Profiler Human XL Cytokine Array Kit using equal amounts of cell lysates from ECs cultured alone (EC only) and in co-cultures with pericytes (EC co). Representative array blots are shown after a short exposure time of 3 min (**b**) and after long exposure time of 25 min (**d**). Bar graphs show the average signal intensities of the framed spots on the array blots and include measurements of two independent experiments at high (**a**) and low (**c**) exposure times, respectively. The legends on the right of the array blots denote the proteins as numbered in the respective blots. Analysis was performed with the image processing software ImageJ (including background subtraction). Experiment was performed twice with independent samples.

To validate some of our gene expression results also on the protein level (Supplementary Figure S10), we performed a membrane-based antibody array. Since we observed a high number of DRGs related to inflammation, we decided to use the Proteome Profiler Human XL Cytokine Array Kit, which detects the expression of 105 proteins. This membrane-based antibody array detects the relative protein expression levels by means of chemiluminescence, therefore, we used two different exposure times (3 min and 25 min) to capture more and less abundantly expressed proteins. Among the highly expressed proteins, co-culture does not seem to induce big changes, with the only exception of epidermal growth factor (EGF), which was downregulated by 81% (Figure 7a and Table 3). However, regulated proteins detected following prolonged exposure include different cytokines (IL-1-α, IL-1-β, IL-3) and chemokines of the CXC-motif ligands CXCL1, CXCL5, CXCL10, CXCL11, CXCL12, CXCL8 (Figure 7c,d; Table 3). In EC-PC co-cultures, a strong modulation in IL binding proteins was observed with decrease of 100% in IL-18-BP and an increase of 83% in IL-1-R4 (Figure 7d and Table 3). Interestingly, in co-cultured cells hepatocyte growth factor (HGF) increased by 164% and PDGF-AB/-BB by 31%. We also observed a 44–50% decrease in expression of granulocyte-macrophage colony-stimulating factor (CSF-2 and CSF-3) and kallikrein-3. Results are also depicted in Table 3, together with a color-code denoting if the detected protein expression levels correspond with the mRNA levels detected by the microarray.

## 4. Discussion

In this study, we present a fully human in vitro model for the blood brain barrier (BBB) by co-culturing the cerebral microvascular endothelial cell line (hCMEC/D3) with primary brain vascular pericytes (hBVP) grown on opposite sides of a porous transwell membrane to maintain basolateral contact through the 0.4 µm pores. Studies with co-culture models often make use of pure animal or a mixture of animal and human cell types, which may be of a poor predictive value for translation into human physiology [28,29]. It is essential to understand the mechanism of barrier function in fully human models of the BBB as there are key differences in the morphology and function between animal and human cells in the brain [29]. Although cell-cell junctional units are complex, the 2D EC-PC model used provides a simple and inexpensive setup to explore the relative contribution of single cell types in regulating BBB function [9,29]. More recently, complex 3D models and spheroids with human induced pluripotent stem cells have been developed [22,28,29]. However, spheroids do not allow for TEER measurements, and the assembly of such models is expensive and complex. 

The observed increase of barrier function in endothelial cells (ECs) cultured with pericytes (PCs) on the opposing side of a transwell insert is in agreement with several previous studies [11,12,21,30]. The fact that the permeability to higher molecular weight dextrans (40 kDa) is decreased in presence of PCs indicates that trans-cellular transport pathways are affected in our co-culture model as such large molecules most probably travers the endothelium via non-specific transcytosis [31]. This observation is in agreement with a previous study showing increased trans-cellular pathways in pericyte-deficient mice [1,2]. The observed increase in TEER and the decreased permeability to 4 kDa dextrans further imply that PCs affect para-cellular transport of ECs by potentially inducing and/or maintaining the function of junctional complexes between neighboring cells [32,33]. Given the small hydrodynamic radius of 4 kDa FITC dextran of 1.4 nm, passage of this tracer via the paracellular pathway is feasible [34]. In addition, even though transporters and channels in the cell membrane also affect the ohmic resistance of a cell layer, at low frequency measurements the contribution of cellular junctions predominates, which infers that cellZcope measurements largely reflect paracellular transport characteristics [35].

Our findings on EC-barrier function using the cellZcope instrument were comparable to those observed using xCELLigence ECIS system, which allows overall assessment of various aspects of cell behavior including adhesion and barrier strength in EC monolayers grown directly on gold-plated electrodes. The xCELLigence electrodes can measure acute temporal changes in barrier resistance across EC monolayer as well as paracellular and basolateral resistance and is more sensitive for assessing barrier function in cell lines [36]. However, the xCELLigence system does not allow the co-culture of cells with basolateral contact for barrier integrity measurements, as was possible with cellZcope.

To better understand the mechanisms involved in the strengthening of endothelial barrier function by PCs, we performed gene expression analysis on co- and mono-cultured ECs. To the best of our knowledge, this is the first study comparing the transcriptomic profile of ECs cultured alone and ECs co-cultured with PCs on the opposite side of a transwell insert with basolateral surface of both cells in direct contact with each other. In a previous transcriptomic study in ECs, the ECs and PCs are co-cultured in a non-contact model (EC’s on membrane and PCs on the culture well surface), thus allowing only contact with soluble factors [37]. 

### 4.1. Effect of Co-Culture on Inflammatory Profile of ECs

In the present study, the most striking change observed in the transcriptomic analysis was the downregulation of several inflammatory cytokines in ECs co-cultured with PCs compared to EC monocultures. This was also highly reflected in the results from pathway analysis, with a great number of regulated pathways in co-cultured cells related to inflammation. This includes obvious inflammatory pathways induced by inflammatory mediators like interferons, interleukins or TNF-α, but also receptor of advanced glycation end products- (RAGE-) and FOS-related antigen- (FRA-) pathways comprise an inflammatory component [38,39]. Apart from the so-called homeostatic cytokines (such as CXCL12, CCL20 or XC3XL), which are expressed at normal or even high levels under physiologic conditions [40], pro-inflammatory cytokines are secreted at high concentrations in response to different inflammatory stimuli and have been shown to exert negative effects on the BBB [24,41,42]. In the present study, the decreased pro-inflammatory profile of ECs co-cultured with PCs is one possible reason for improved barrier function in co-cultures [24]. The fact that most of the transcriptionally regulated cytokine mRNAs also show a corresponding trend in the protein expression highlights the significance of the observed changes. It further confirms the reliability of our microarray data by demonstrating that the transcriptional regulation also triggers changes in corresponding protein levels. Compared to most other cytokines, we did not observe congruent changes in gene and protein expression of CXCL12, which may be due to posttranscriptional regulation commonly observed in cytokines [43]. Our results indicate that co-culture with PCs contributes to maintaining a low inflammatory profile by downregulation of several pro-inflammatory cytokines on the transcriptional as well as on the protein level. 

The above notion is further supported by the fact that many different interleukins and chemokine CXC-motif ligands, which are upregulated following ultrasound and infection induced barrier disruption [23,42,44], were downregulated in ECs co-cultured with PCs. Moreover, in co-cultured ECs, we observed a decrease in IL-18-binding protein and an increase in IL-1-R4. Since IL-18 is protective against BBB-disruption [45], a decrease in IL-18-BP would increase IL-18 availability and result in increased protective actions on the BBB. With regard to IL-1-R4 (also called ST2), which is a decoy receptor for IL-33 and blocks its function [46,47], upregulation in its expression may protect BBB against IL-33 mediated EC insult, activation and microglia-mediated neuroinflammation. We also observed a decrease in expression of granulocyte-macrophage colony-stimulating factors (CSF-2 and CSF-3) and kallikrein-3, three key inflammatory players [48,49]. Both CSFs and kallikrein-3 are known to negatively influence BBB dynamics and integrity; hence their downregulation in ECs co-cultured with PCs would reflect their protective role in maintaining EC integrity.

### 4.2. Soluble Mediators and Cell Surface Proteins

In ECs co-cultured with PCs, hepatocyte growth factor (HGF) increased by 164%, platelet-derived growth factor-AB/-BB (PDGF-AB/-BB) by 31%, whereas EGF was decreased by 81%. Since HGF and PDGF-AA/-BB have been shown to improve BBB integrity and function [50,51], whereas EGF increases BBB permeability [52], the PC induced changes reflect improvement in EC barrier. Different soluble factors, including PDGF-B, vascular endothelial growth factor (VEGF), transforming growth factor beta (TGF-β), basic fibroblast growth factor (FGF-2) and angiopoietin 1 and 2, have been shown to contribute significantly to the paracrine signaling between ECs and PCs [51]. The importance of PDGF-B with regard to EC-PC interaction has been demonstrated in numerous in vitro as well as in vivo studies [51]. Our observation of increased PDGF-AB/-BB protein expression in co-cultured ECs is in line with the relevance of this growth factor in EC-PC crosstalk. VEGF, a potent stimulator of angiogenesis under physiological but also pathological conditions [53], is a well-known inducer of vascular permeability at high concentrations [54,55]. The observed decrease of VEGF-C, but no change in VEGF-A and VEGF-B expression in co-cultured ECs, therefore, depicts a possible mechanism for barrier induction in co-culture, by reducing autocrine BBB disruption. Interestingly, it has been observed that VEGF-C/VEGFR2 signaling in PCs leads to vessel destabilization by antagonizing PDGFR-β signaling and depleting PC coverage of the blood vessels [56], which presents another potential explanation on how downregulation of VEGF-C could have beneficial effects on endothelial barrier. 

The reduced expression of VEGF-C might also be linked to the downregulation of the cellular receptor for lysophosphatidic acid (LPAR1), as LPAR1 is known to mediate VEGF-C induction by LPA, thereby promoting angiogenesis [57]. The impact of LPA on BBB is controversial and thought to be tissue-dependent [58]. However, in vitro and in vivo experiments clearly show that LPA signaling diminishes BBB function [25]. The reduced expression of LPAR1 in co-cultured ECs might therefore also contribute to the barrier improving effects by suppressing angiogenic activities of ECs. Another cell surface protein that is downregulated in EC co-cultures on mRNA as well as protein level (Supplementary Figure S12) is the NOTCH ligand Jagged 1. Our finding is further supported by Kang et al. [14], who observed downregulation of Jagged 1 on the protein level in ECs co-culture with PCs in the same model as we used in the present study (indirect EC-PC co-culture with contact through a permeable membrane). NOTCH 1/Jagged 1 signaling is involved in juxtacrine communication between ECs and mural cells and plays an important role during vascular development [59], whereas increased Jagged 1 levels in vessels are linked to barrier disruption via NOTCH and VE-cadherin/beta-catenin signaling [27]. Additionally, the well-established barrier disrupter TNF-α upregulates Jagged 1 expression [14]. A cellular receptor potentially supporting barrier function in our co-culture model is leptin receptor (LEPR). Deficiency in leptin receptor induces BBB dysfunction in mice [26]. Hence, the observed decrease in Jagged 1/increase in LEPR expression in EC co-cultured with PC may contribute to the improved barrier function in our model. 

### 4.3. Extracellular Matrix Regulation

The high impact of PC-EC co-culture on the regulation of ECM proteins is apparent from the pathway analysis results, as the two most significantly enriched pathways are linked to regulation of ECM. This could explain why the positive effect of co-culture is not mimicked by basolateral treatment with PC conditioned media (PCM), since immediate contact of the two cell types and the interaction with their extracellular environment is required. This contention is supported by the findings by Hartmann et al. who demonstrated that ECs cultured on pericyte-derived ECM form a tighter barrier than when cultured on their own matrix [60]. Reinforcing this hypothesis, connexin 43, a major gap junction protein that is known to be involved in the formation of direct contacts between ECs and PCs [61,62], is upregulated in PC co-cultures (unpublished data). Connexin 43 has been shown to play an important role in barrier function by co-localizing with TJ proteins, and the deletion of this protein results in severe barrier dysfunction [63,64]. 

Transforming growth factor beta (TGF-β) signaling is highly regulated upon co-culture, as is evident from the pathway enrichment analysis, showing the most significantly regulated pathway to be ‘TGF-β regulation of ECM’. We also observed favorable changes in PECAM-1/CD31, SERPIN E1/PAI-1, Endoglin, PLAUR/uPAR/CD87, all TGF-β associated factors known to preserve EC barrier function. TGF-β superfamily ligands are a versatile group of different secreted factors including bone morphogenetic proteins (BMPs), activins, inhibins, nodals as well as growth and differentiation factors [65]. They control several aspects in the developing organism and play an important role in tissue homeostasis in the adults by regulating different function such as cell proliferation, differentiation as well as ECM production. Hence, TGF-β signaling strongly depends on spatial and temporal expression profiles of all involved signaling constituents, which renders a highly complex regulatory system. Several target proteins of the TGF-β pathway include ECM proteins, which are themselves implicated in TGF-β regulation, mainly by activating latent forms of TGF-β subtypes [66]. Due to the complex nature of TGF-β signaling, a thorough analysis of regulated proteins involved in this pathway is beyond the scope of this study. 

### 4.4. Junctional Proteins

In ECs co-cultured with PCs, we observed no change in claudin-5 or Zonula occludens proteins (ZO-1 to 3), but a decrease in occludin and claudin-1 gene expression, compared to ECs alone. Moreover, we observed a decrease in claudin-5 protein expression (Supplementary Figure S13). Junctional proteins, such as claudin-5 and occludin, play a critical role in regulating BBB permeability. However, increasing evidence suggests that BBB permeability can be influenced by junctional protein-independent mechanisms. In Claudin-5 knock out mice, the size-selective loosening of BBB is observed with change in BBB morphology [67]. Reduced barrier function under hypoxic conditions is induced by TJ rearrangement, while the protein expression remained stable or even increased slightly, however non-significantly [68]. Similarly, BBB damage in the early phase of ischemic stroke leads to a claudin-5 re-localization, but no change in overall protein levels is observed [69]. Interestingly, claudin-1 dependent destabilization of BBB has been observed in chronic stroke [70]. Moreover, claudin-1 can interfere with claudin-5 as its appearance in tight junction complex reduces claudin-5 strands and claudin-5/ZO-1 interaction, thereby limiting its incorporation in tight junction complex [70]. Hence the finding of decreased claudin-1 in ECs co-cultured with PCs would positively influence EC barrier. Taken together, these findings support the notion that bare TJ protein expression levels do not always meaningfully reflect barrier integrity, neither on the mRNA nor on the protein level. Notably higher consideration should be attributed to post-translational modifications like phosphorylation status, molecular interactions and cellular localization of TJ proteins [68,69,71]. 

### 4.5. Effects of Pericyte Conditioned Media (PCM) on Endothelial Barrier Function

Crosstalk between ECs and PCs depends on paracrine signaling as well as direct cell–cell interactions. Different studies showed positive effects of PC-conditioned media (PCM) on EC barrier function [12,72]. Our results from PCM experiments, however, indicated no change in barrier properties of EC monolayers by PC-released soluble factors (Supplementary Figure S5). We further used PCM from co- and mono-cultured PCs and did not observe any differential effects between co- and mono-cultured PCM. Potential explanation(s) for the lack of barrier improving effects of PCM on EC function can only be speculated upon. It is feasible that PC-derived factors are present in high concentration gradient between EC-PC basolateral interface. Moreover, active contribution of endoplasmic vesicles in facilitating PC-EC communication and differences in factors generated from apical and basolateral cell surface cannot be ruled out.

Differential expression of several transporters, channels and lipids on the luminal and abluminal side is a well-known feature of ECs and plays an important role in BBB integrity [73]. Various soluble factors have been shown to induce polarized signaling, such as VEGF, histamine, LPA, TGF-β and LPC [74]. The observed differential effects of PCM on the apical and basolateral surface of an endothelial monolayer regarding barrier function can be explained by this apicobasal polarity of ECs. Whereas basolateral PCM treatment did not significantly change barrier function of an already established EC monolayer, it had demolishing effects if applied apically. The fact that conditioned media from SMCs showed similar deteriorating effects leads to the conclusion that the observed effect is not specific to PCs but might rather be explained by the presence of apicobasal polarity of ECs. Similarly, when PCM was applied basolaterally to growing EC monolayer, where the barrier was not yet formed and soluble factors could pass through the permeable transwell membrane, thereby acting on the apical side of ECs, a decrease in TEER was observed. This was accompanied by an increase in permeability to low molecular weight dextran (4 kDa). Both of these results represent an effect on paracellular rather than transcellular permeability (see above). Taken together, these observations from PCM experiments indicate that soluble mediators secreted by PCs seem to play a subordinate role in the process of para-cellular barrier improvement in our model. 

The non-contact co-culture model with PCs grown on the well-plate surface while ECs were cultured in the transwell insert resulted in a decrease in barrier function when compared to an EC monolayer. This experimental set-up reflects the situation of prolonged PCM treatment resulting in decreased barrier function. Hatherell et al. [30] observed a comparable effect by investigating different configurations of co-culture models including ECs, PCs and also astrocytes (ACs). While culturing ECs and PCs together in the indirect co-culture model on a porous membrane increased barrier function, the addition of PCs to the well surface of an EC-AC co-culture led to a significant drop in the measured TEER. Unfortunately, no data with ECs and PCs alone was shown in the non-contact model used. These results are in contrast to other studies, which observed improved barrier properties in the non-contact co-culture model or after PCM treatment [10,11,75]. A potential explanation for the different outcomes may be differences in experimental setup and/or the use of cells from different species.

In the past, the oversimplified and non-physiological model obtained by simply combining PCs and ECs as co-cultures has been used [76,77,78]. Our finding of a reduced barrier function in this direct PC-EC co-culture is a clear indication for the non-physiologic nature of this model. The same effect has also been observed by another in vitro study performed with iPSC-derived pericytes [22]. By fluorescent imaging, they further showed that PCs outcompete ECs for covering the glass surface, such that ECs are forced to overgrow PCs, which most probably leads to barrier deterioration.

In conclusion, our results provide first evidence that in human EC-PC co-cultures, contact on the basolateral surface represents a physiological orientation that is responsible for increased EC barrier function. PCs regulate EC barrier function on the para- as well as transcellular level. Transcriptomic analysis of genes and cytokine proteome arrays support our findings from in vitro barrier studies and highlight that PC induced EC barrier function is associated with decrease in pro-inflammatory cytokine profile, regulation of TGF-β as well as extracellular matrix pathways and multiple permeability improving soluble factors, but not tight junction proteins. Using human PC-EC co-cultures with basolateral contacts may serve as a viable in vitro model for investigating BBB pathophysiology in inflammatory and neurodegenerative disease, as well as for therapeutic drug delivery studies.

## Figures and Tables

**Table 1:** Pathway enrichment analysis of differentially regulated genes (DRGs) between endothelial cell co-cultures and monocultures.

Pathway	Overlap	Adj. *p*-Value
TGF-beta regulation of extracellular matrix	40/565	9.37E-23
Interleukin-1 regulation of extracellular matrix	16/120	2.98E-12
Gastrin pathway	9/44	4.41E-08
TNF-alpha effects on cytokine activity, cell motility, and apoptosis	12/135	5.55E-07
Interleukin-5 regulation of apoptosis	12/144	9.32E-07
Interleukin-2 signaling pathway	26/847	2.30E-06
Immune system signaling by interferons, interleukins, prolactin, and growth hormones	15/280	3.02E-06
Fos-related antigen (FRA) pathway	7/37	3.93E-06
Oncostatin M	14/311	6.16E-05
Interferon alpha/beta signaling	7/64	1.57E-04
Interferon signaling	10/168	2.09E-04
Fibroblast growth factor 1	5/26	2.75E-04
T cell receptor regulation of apoptosis	18/603	3.63E-04
Thymic stromal lymphopoietin (TSLP) pathway	7/90	0.00112
Receptor of advanced glycation end products (RAGE) pathway	6/60	0.00107
Cytokine-cytokine receptor interaction	11/265	0.00138
Hypertrophy pathway	4/20	0.00182
Activator protein 1 (AP-1) transcription factor network	6/70	0.00219
Epidermal growth factor receptor 1 (EGFR1) pathway	8/152	0.00336
Cytosolic tRNA aminoacylation	4/24	0.00330
Type II interferon signaling (interferon-gamma)	5/50	0.00430
Ataxia-telangiectasia mutated (ATM)-dependent DNA damage response	6/82	0.00441
TNF-like weak inducer of apoptosis (TWEAK) regulation of gene expression	4/27	0.00465
Follicle-stimulating hormone (FSH) regulation of apoptosis	10/263	0.00475
Folate metabolism	5/63	0.0110

Analysis was performed using NCATS BioPlanet on the Enrichr website. Second column describes number of regulated genes compared to total number of genes in the pathway and the *p*-value adjusted for multiple testing is noted in the last column. DRGs were determined based on a fold change (FC) cut-off of 1.5 (log2FC ± 0.59) and an FDR *p*-value of 0.05.

**Table 2:** List of differentially regulated genes (DRGs) potentially linked to barrier function [23,24,25,26,27].

Gene	Gene Description	log2 FC (co- vs. Monoculture)	FDR *p*-Value
Soluble mediators			
CXCL8	Chemokine (C-X-C motif) ligand 8	−3.7	1.68E-07
CXCL5	Chemokine (C-X-C motif) ligand 5	−2.2	0.0009
CXCL6	Chemokine (C-X-C motif) ligand 6	−1.9	0.036
CXCL10	Chemokine (C-X-C motif) ligand 10	−1.5	0.0045
IL1B	Interleukin 1 beta	−2.4	0.0003
IL1A	Interleukin 1 alpha	−1.4	0.0021
IL32	Interleukin-32	−1.7	0.0002
VEGFC	Vascular endothelial growth factor C	−1.3	0.0016
TGFB2	Transforming growth factor beta 2	1.1	0.016
BMP4	Bone morphogenetic protein 4	1.5	0.0048
BMP6	Bone morphogenetic protein 6	1.4	0.0021
			
Junctional Proteins			
CLDN1	Claudin-1	−2.0	4.69E-05
OCCL	Occludin	−1.1	0.034
			
ECM Proteins			
ADAMTS6	ADAM metallopeptidase with thrombospondin type 1 motif 6	−1.3	0.0008
LAMB3	Laminin beta 3	−1.9	0.0008
SERPINE1	Serpin peptidase inhibitor, calde E (nexin, plasminogen activator inhibitor, type 1)	−1.6	0.0012
PLAU	Plasminogen activator, urokinase	−1.5	0.0019
LTBP1	Latent transforming growth factor beta binding protein 1	−1.1	0.0022
EFEMP1	EGF containing fibulin-like ECM protein1	0.86	0.0125
SERPINA3	Serpin peptidase inhibitor, calde A (alpha-1 antiproteinase, antitrypsin)	1.6	0.0252
BGN	Biglycan	1.5	0.0348
			
Cell surface proteins			
JAG1	Jagged 1	−1.2	0.020
LPAR1	Lysophosphatidic acid receptor 1	−1.3	0.0009
LEPR	Leptin receptor	1.1	0.0092

Log2 Fold Changes (log2FC) of gene expression levels in co-cultured vs. mono-cultured endothelial cells and adjusted *p*-values are depicted in the third and fourth column, respectively.

**Table 3:** List of differentially expressed proteins using a cytokine proteome array.

Protein	Array Coordinates	Protein Regulation	mRNA Regulation
CXCL1	D1	−55%	down (ns)
CXCL5	C2	−34%	down
CXCL8	E4	−25%	down
CXCL10	F10	−61%	down
CXCL11	F11	−59%	nr
CXCL12	H12	−31%	up (ns)
IL-1-a	D8	−64%	down
IL-1-b	D9	−34%	down
IL-1-Ra	D10	−50%	nr
IL-1-R4	I3	+83%	up
IL-3	D12	−100%	nr
IL-18-BP	E12	−100%	nr
PDGF-AB/-BB	H4	+31%	nr
HGF	D3	+164%	nr
MIC-1	C10	−47%	nr
CSF-2	C11	−46%	nr
CSF-3	C9	−44%	nr
Kallikrein 3	F12	−53%	nr
PECAM1 (CD31)	J4	+14%	nr
SERPIN E1	I1	+16%	down
Pentraxin 3	H5	−27%	down
EGF	B10	−81%	nr
Endoglin	C3	+10%	up (ns)
PLAUR	I10	−11%	down (ns)
DPPIV	B9	−17%	nr
ICAM-1	D4	+16%	nr
MIF	G7	+20%	nr
Emmprin	B11	+7%	nr

List of differentially expressed proteins using a cytokine proteome array (membrane-based, chemiluminescent detection). Array coordinates are shown in the second column for localization of spots in Figure 7b,d. Column 3 denotes the observed change in protein expression in co-cultures in relation to protein levels in mono-cultures. Analysis was performed with the image processing software ImageJ (including background subtraction). The last column denotes regulation of the corresponding mRNA levels upon co-culture, as was determined by the microarray (upregulated (up); downregulated (down); not regulated (nr); ns: non-significantly with a *p*-value < 0.05, but FDR *p*-value > 0.05). Matching transcriptomic and proteomic results are color-coded in green, opposing results are highlighted in orange.

## Data Availability

All data supporting the findings of this study are available within the article and its supplemental data file or from the corresponding author upon reasonable request.

## Supplementary Materials

The following are available online at https://www.mdpi.com/article/10.3390/cells10040963/s1, Supplementary Figure S2: Measuring barrier function with the xCELLigence system, Supplementary Figure S2: Effect of pericyte (PC) cell number on improvement of barrier function; Supplementary S3: Real-time TEER measurement of mono- and co-cultures of endothelial cells (ECs) and pericytes (PCs); Supplementary Figure S4: Addition of pericytes (PCs) to an established endothelial (EC) monolayer; Supplementary Figure S5: Effect of basolateral pericyte-conditioned media (PCM) on endothelial barrier function; Supplementary Figure S6: Effect of non-contact co-culture on barrier function; Supplementary Figure S7: Conditioned media from co-cultured and mono-cultured pericytes (PCs); Supplementary Figure S8: Measurements of endothelial barrier function after apical treatment with conditioned media from smooth muscle cells (SMC-CM); Supplementary Figure S9: Effect of pericyte conditioned media (PCM) on cell viability; Supplementary Figure S10: Workflow of sample collection for the microarray and proteome array; Supplementary Figure S11: Principle Component Analysis (PCA); Supplementary Figure S12: Jagged-1 expression in co-culture endothelial cells (ECs); Supplementary Figure S13: Claudin-5 expression decreases in co-culture endothelial cells (ECs); Supplementary Table S1: Top ten upregulated genes in co-cultured endothelial cells.; Supplementary Table S2: Top ten downregulated genes in co-cultured endothelial cells
