# Supplementary material for: Transcryptomic Analysis of Human Brain-Microvascular Endothelial Response to -Pericytes: Cell Orientation Defines Barrier Function
Source: Cells. 2021 Apr 20;10(4):963. doi: 10.3390/cells10040963 (PMC8074760; doi:10.3390/cells10040963)
Supplement: Supplementary file 1 [file cells-10-00963-s001.zip › cells-1183691-supplementary.pdf]

cells

MDPI

Article

Transcryptomic Analysis of Human Brain -Microvascular Endothelial Response to -Pericytes: Cell Orientation Defines Barrier Function

Lisa Kurmann[1], Michal Okoniewski[2], Omolara O. Ogunshola[3], Brigitte Leeners[1], Bruno Imthurn[1] and Raghvendra K. Dubey[1,4]*

1 Department of Reproductive Endocrinology, University Hospital Zurich; lisa.kurmann@usz.ch, brigitte.leeners@usz.ch, bruno.imthurn@uzh.ch, raghvendra.dubey@usz.ch
2 ID Scientific IT Services, ETH Zurich, michal.okoniewski@id.ethz.ch
3 Institute of Veterinary Physiology, Vetsuisse Faculty and Zurich Center Integrative Physiology (ZIHP), University of Zurich; larao@access.uzh.ch
4 Department of Pharmacology & Chemical Biology, University of Pittsburgh, Pittsburgh, PA, USA
* Correspondence: raghvendra.dubey@usz.ch

Supplementary Material / data

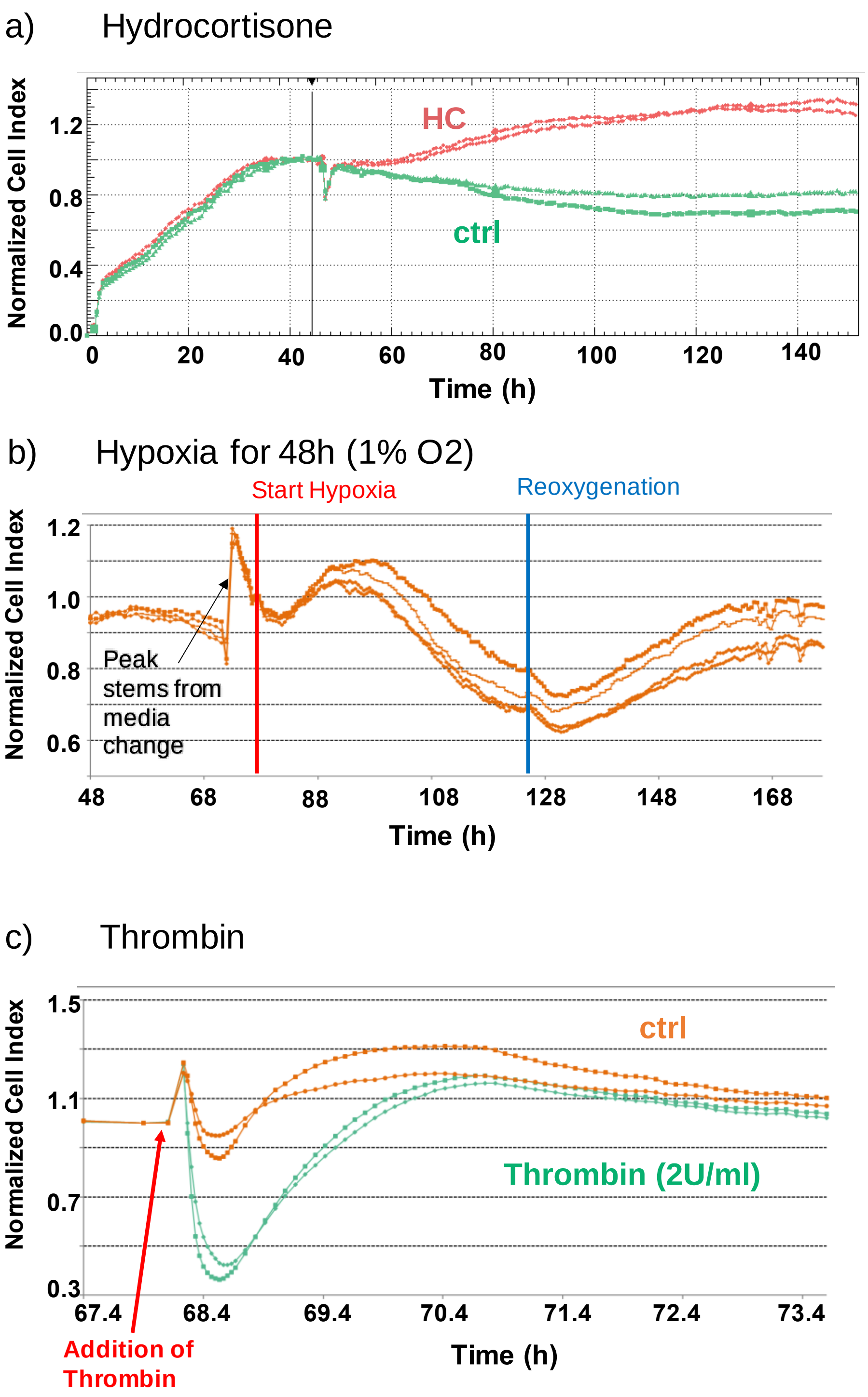

Suppl. Figure 1: Measuring barrier function with the xCELLigence system.

Endothelial cells were seeded into eplates at a density of 100’000 cells / cm^2. After a stable baseline was established, cells were either treated with hydrocortisone (1 µg/ml) **(a)**, Thrombin 2 U/ml **(c)** or added to a hypoxia chamber (1% O_2, 5% CO_2) for 48h before reoxygenation **(b).** Impedance was recorded in real-time and converted to cell index by the xCELLigence RTCA software. Normalized cell index is shown for all graphs.

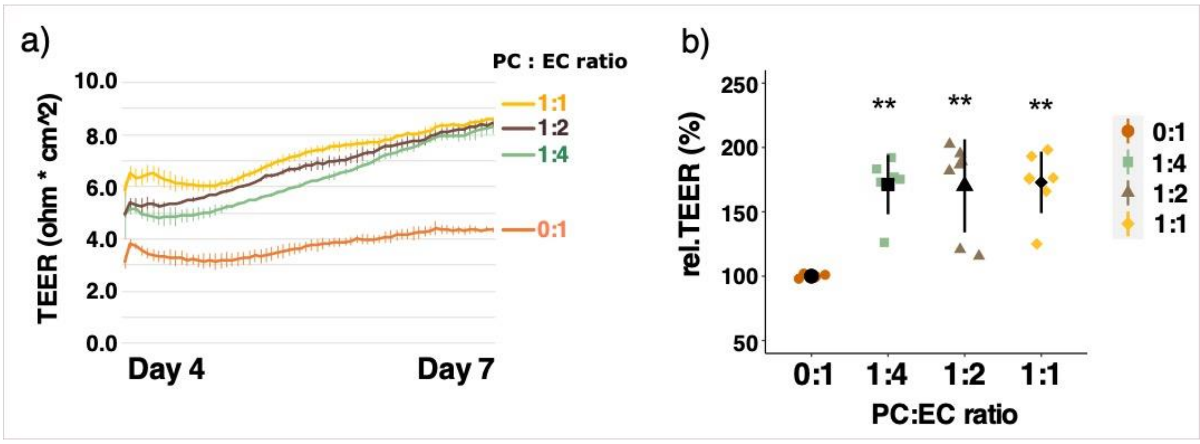

Suppl. Figure 2: Effect of pericyte (PC) cell number on improvement of barrier function.

PCs were seeded at different ratios to endothelial cells (ECs) on the basolateral side of a transwell insert, while ECs were added to the apical side. Results are shown as representative real-time TEER measurements **(a)** or as a summary after 7 days in culture **(b).** Experiments were performed 3 times in duplicates or triplicates and data represent mean +/- SD. ** $P < 0.01$.

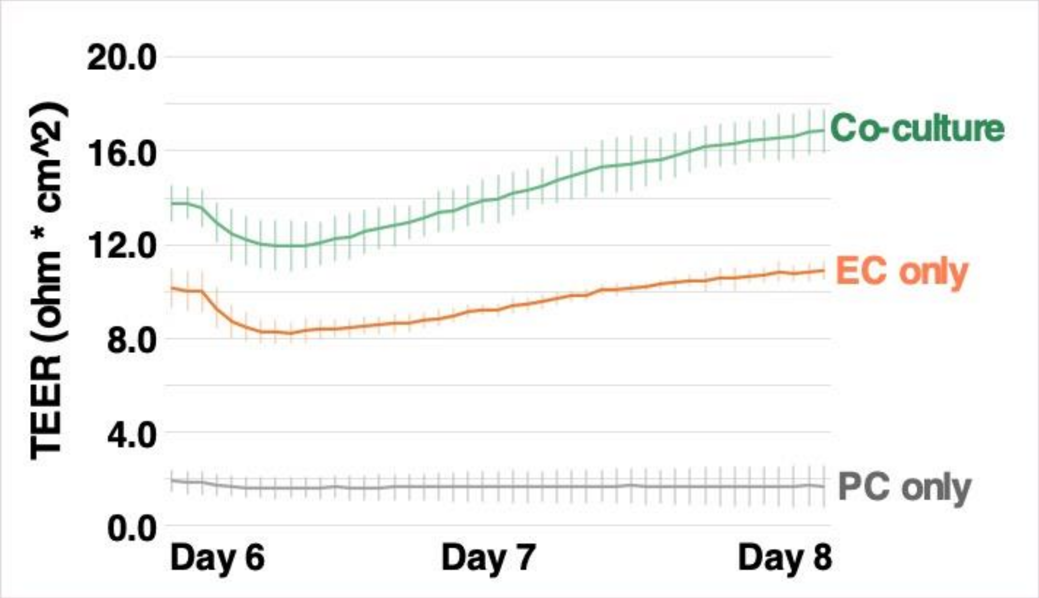

Suppl. 3: Real-time TEER measurement of mono- and co-cultures of endothelial cells (ECs) and pericytes (PCs).

ECs and PCs were cultured on transwell inserts alone or in co-culture. PCs plated on the basolateral side of the insert and ECs on the apical side. Experiment was performed two times in duplicates and representative real-time TEER measurements are shown (means +/- SD).

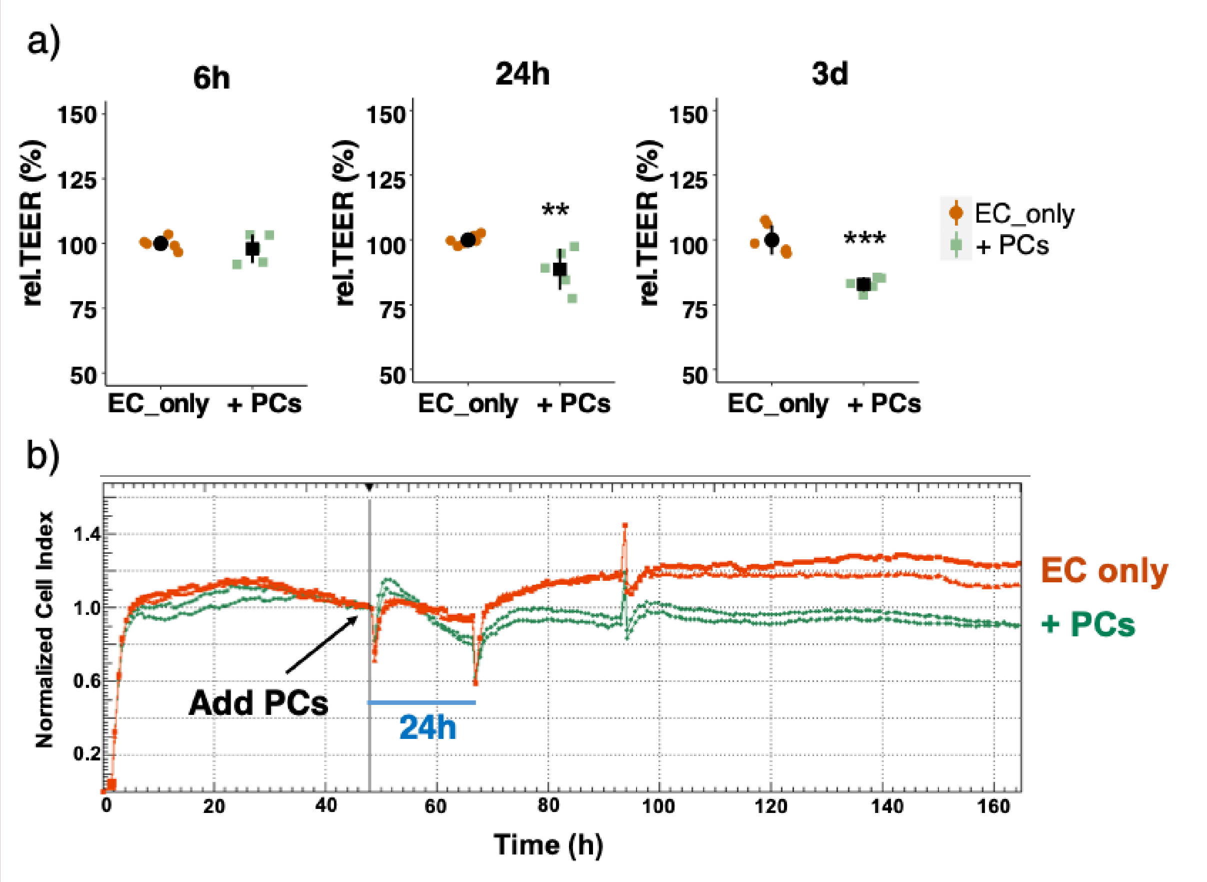

Suppl. Figure 4: Addition of pericytes (PCs) to an established endothelial (EC) monolayer.

ECs were cultured on transwell inserts. After 5 days, PCs (7'500 / insert) were added to the apical chamber. Relative TEER measurements are shown 6h, 24h and 3d after PC addition **(a)**. Experiments were performed at least three times in duplicates and data represent mean +/- sd. ** $P < 0.01$, *** $P < 0.001$. Representative experiment from barrier function measurements using the xCELLigence system **(b)**. PCs were added to a confluent monolayer of ECs grown in eplates. Relative cell index is shown in real-time.

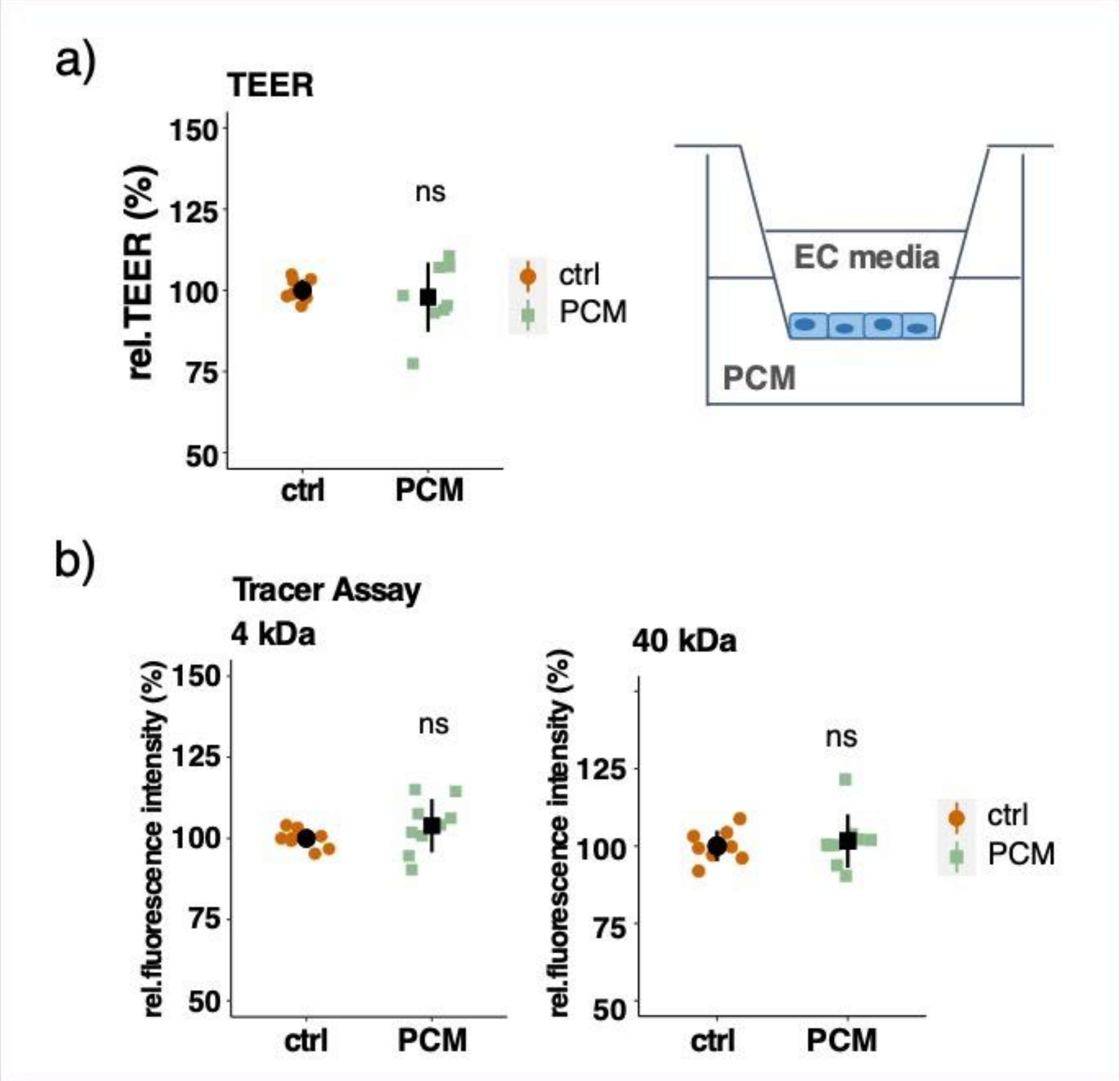

Suppl. Figure 5: Effect of basolateral pericyte-conditioned media (PCM) on endothelial barrier function.

Endothelial cells were cultured on transwell inserts. After 5 days, PCM was added to the basolateral side for 3 days. **(a)** TEER measurements, **(b)** macromolecular tracer assay with FITC-labeled dextran of 4 and 40 kDa. Experiments were performed at least three times in triplicates and data represent mean +/- sd.

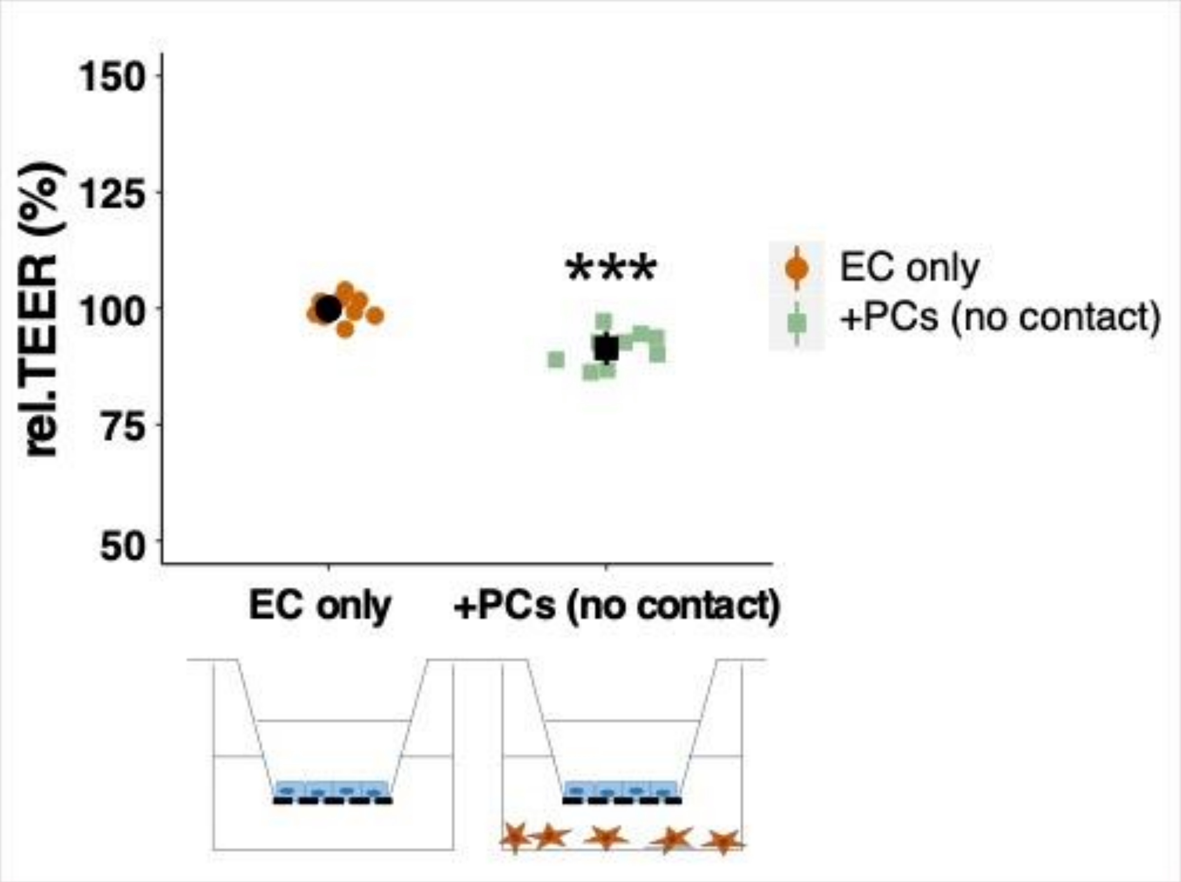

Suppl. Figure 6: Effect of non-contact co-culture on barrier function.

Endothelial cells (ECs) were plated in transwell inserts and pericytes grown on the bottom of the well-plate without contact to ECs. TEER was measured after 7 days in culture. Experiments were performed at least three times in triplicates and data represent mean +/- sd. *** $P < 0.001$.

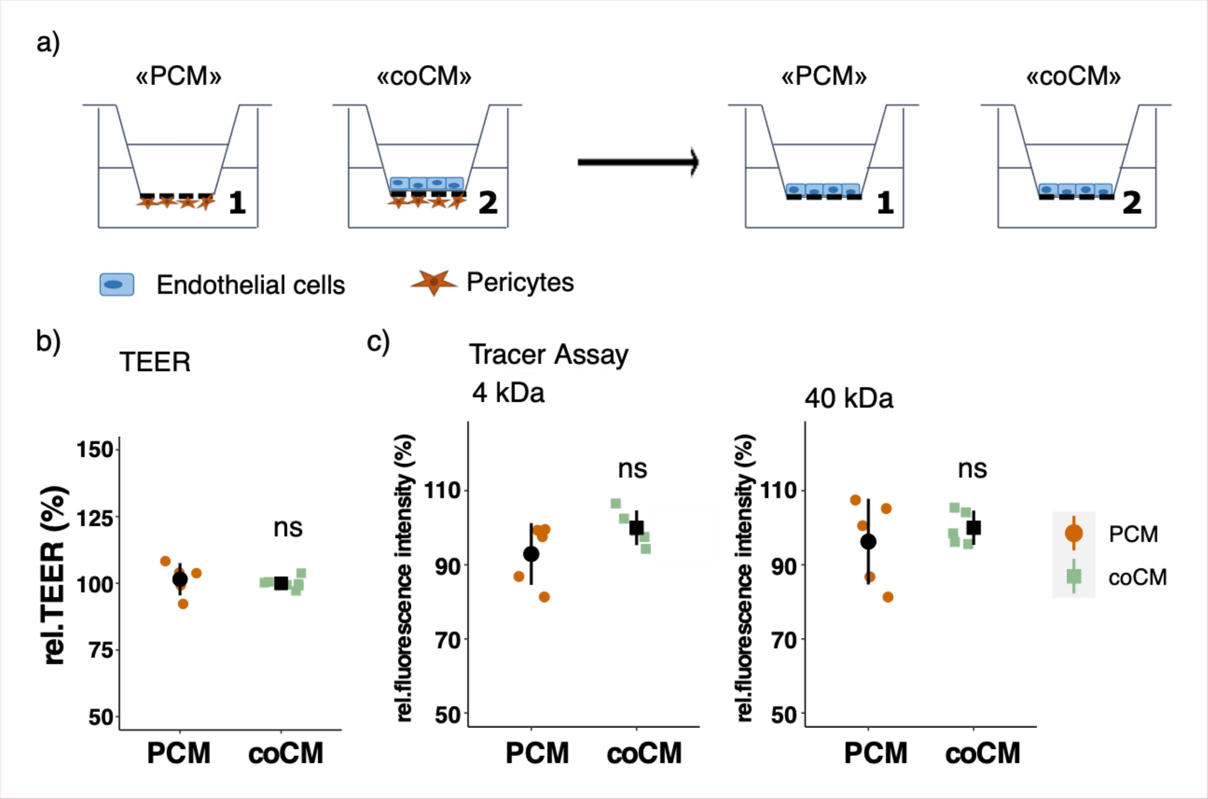

Suppl. Figure 7: Conditioned media from co-cultured and mono-cultured pericytes (PCs).

PCs were cultured on the basolateral side of transwell inserts with or without endothelial cells (ECs) on the apical side for 5 days, before the inserts were taken out. Fresh inserts containing established EC monolayers were added to the same wells **(a)** and barrier function was assessed after 3 days of CM treatment by means of TEER measurements **(b)** and FITC assays with 4 kDa and 40 kDa FITC dextran **(c).**

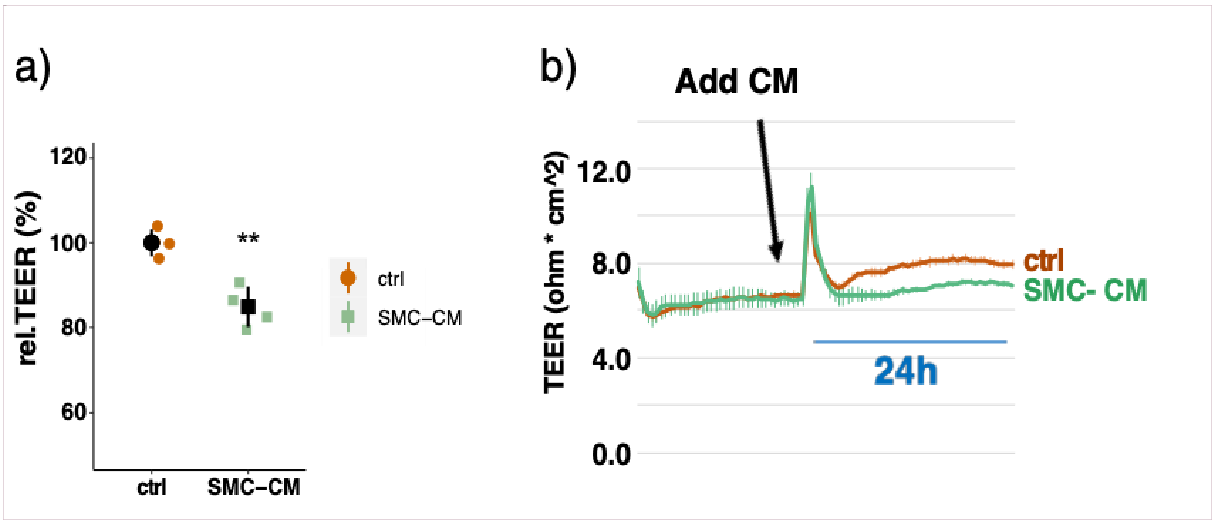

Suppl. Figure 8: Measurements of endothelial barrier function after apical treatment with conditioned media from smooth muscle cells (SMC-CM).

Endothelial cells were cultured on transwell inserts. After barrier function was established (6-7 days), SMC-CM was added to the apical side for 24h **(a).** Representative real-time measurements with the cellZcope instrument **(b).** Experiments were performed two times in duplicates and data represent mean +/- sd. ** $P < 0.01$.

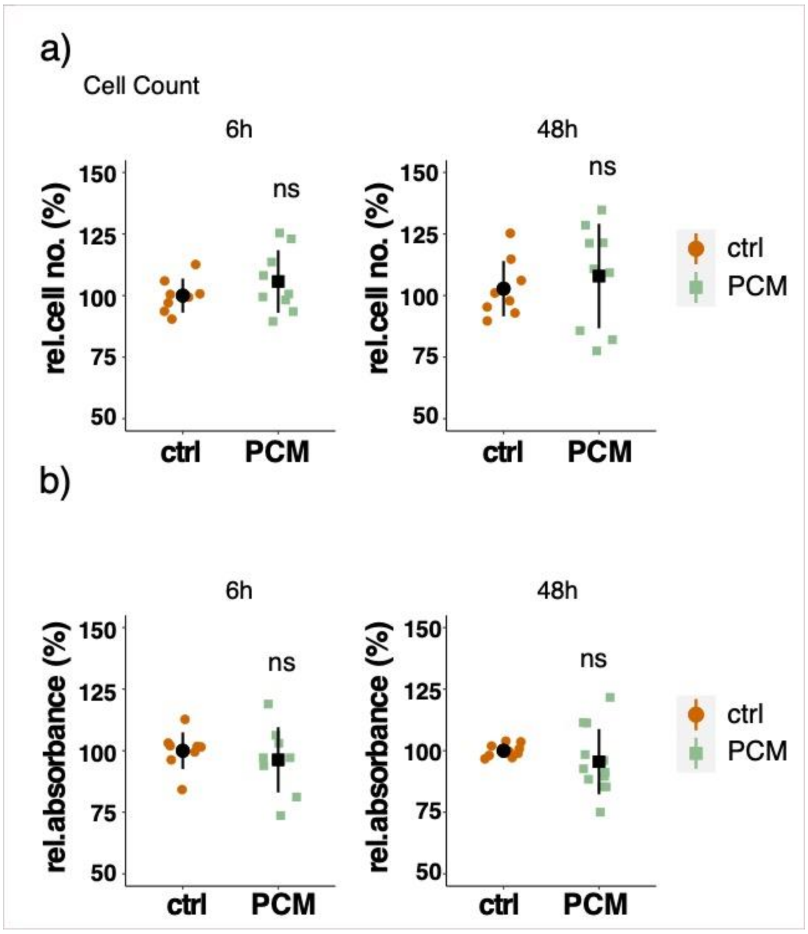

Suppl. Figure 9: Effect of pericyte conditioned media (PCM) on cell viability.

Confluent endothelial monolayers were treated with PCM or control for 6h and 48h, before cell number was assessed by cell-counting with a coulter counter **(a)** or cells were fixed and stained with Crystal Violet Stain (CVS) **(b).** Experiments were performed 3 times in triplicates and data represent mean +/- sd. ns $P > 0.05$, compared to ctrl.

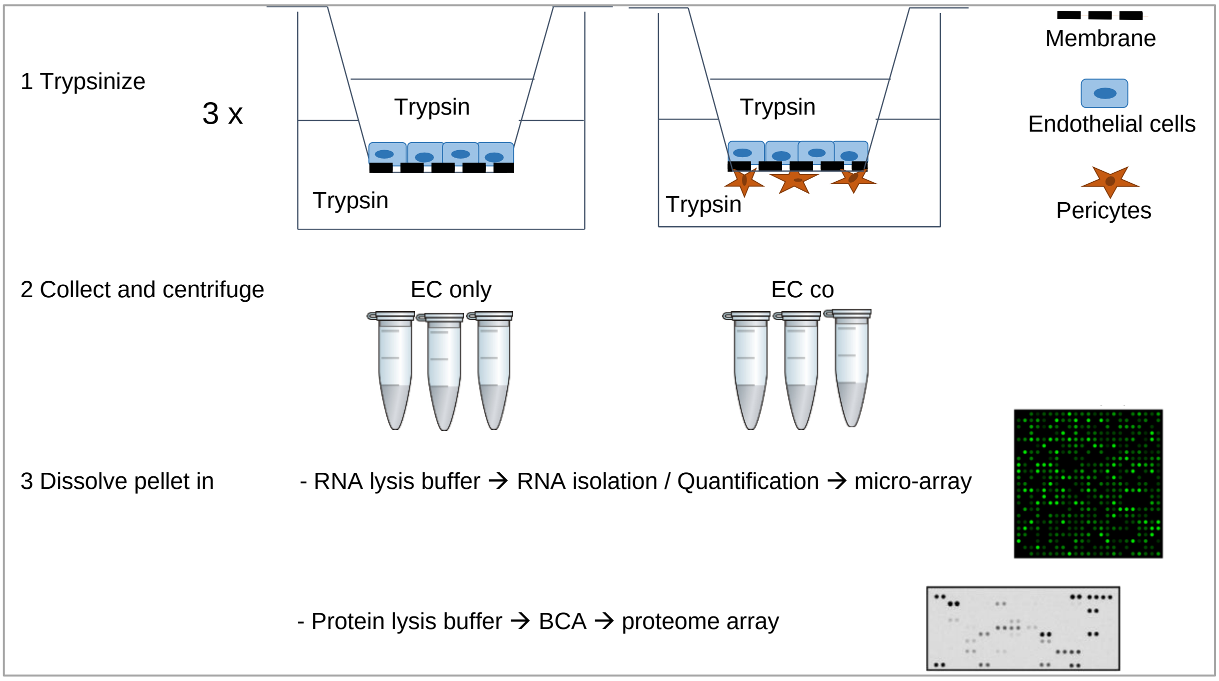

Suppl. Figure 10: Workflow of sample collection for the microarray and proteome array.

ECs were grown on permeable transwell inserts alone or in co-culture with pericytes on the opposite sides of the transwell membrane. After 7 days in culture, cells were trypsinized and centrifuged. The pellet was lysed in RNA lysis buffer (microarray) or in protein lysis buffer (proteome array). Before microarray, RNA was isolated and quantified. For the proteome array, protein quantification was carried out by bicinchoninic acid (BCA) analysis.

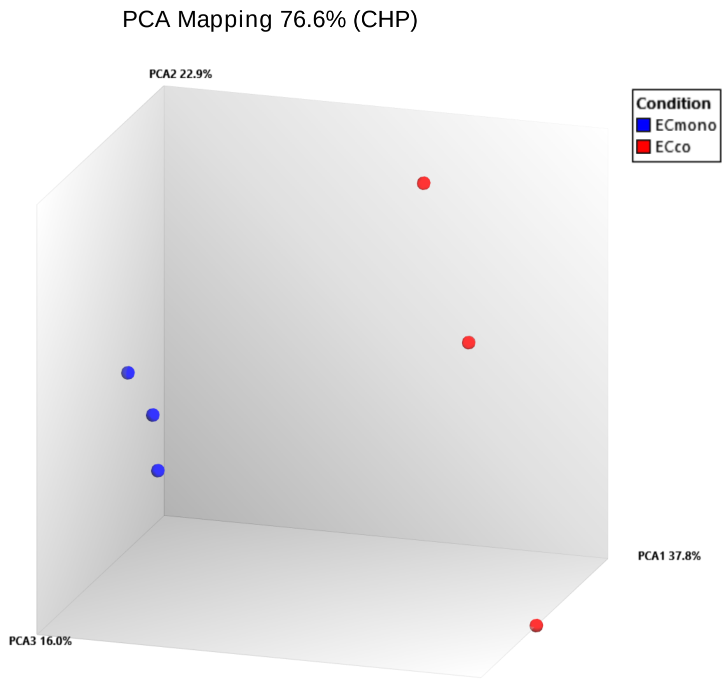

Suppl. Figure 11: Principle Component Analysis (PCA)

PCA shows a clear separation between the samples from endothelial mono cultures (blue) and co-cultures (red). PCA was automatically performed by the Transcriptome Analysis Console (TAC).

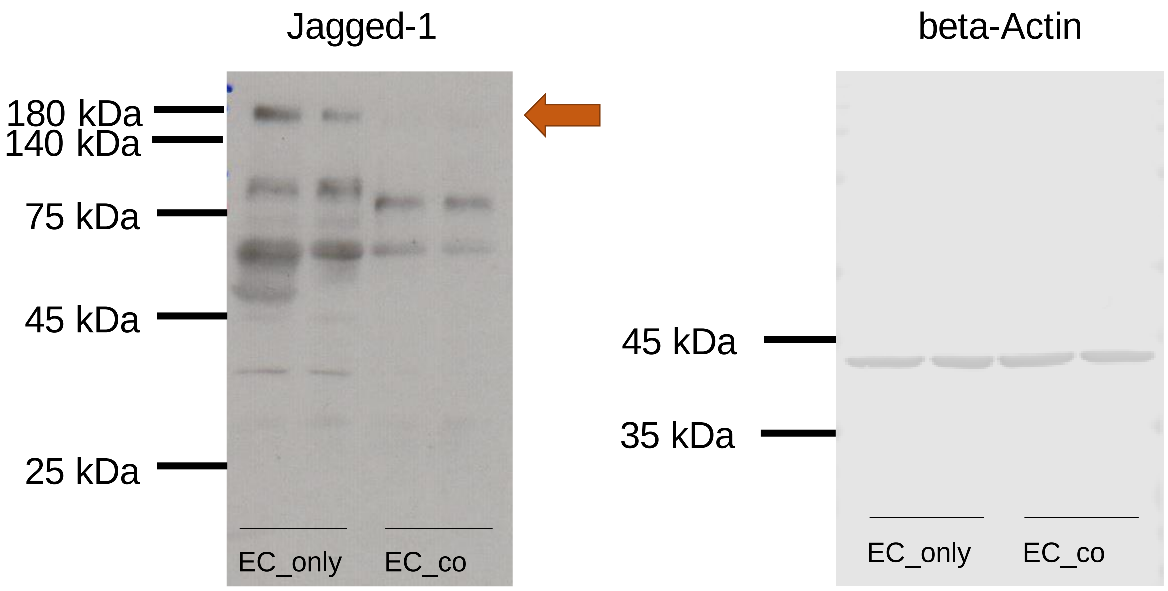

Suppl. Figure 12: Jagged-1 expression in co-culture endothelial cells (ECs).

ECs were cultured on transwell inserts alone (EC_only) or in co-culture with PCs on the abluminal surface (EC_Co) for 7 days before they were trypsinized and lysed for Western blotting. Immunoblots for Jagged-1 (Cellsignalling, #2155, predicted molecular weight: 180kDa) (red arrow) and for beta-actin (Sigma Aldrich, #A5441, predicted molecular weight: 42 kDa) are shown.

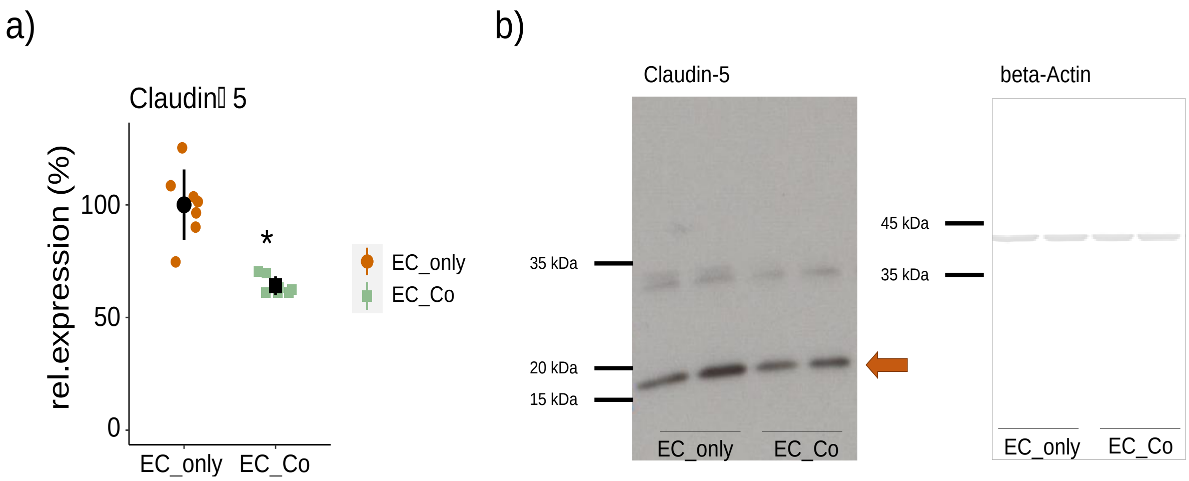

Suppl. Figure 13: Claudin-5 expression decreases in co-culture endothelial cells (ECs).

ECs were cultured on transwell inserts alone (EC_only) or in co-culture with PCs on the abluminal surface (EC_Co) for 7 days before they were trypsinized and lysed for Western blotting. A representative immunoblot for claudin-5 (Invitrogen, #35-2500, predicted molecular weight: 18-23 kDa) (red arrow) is shown with beta-actin (Sigma Aldrich, #A5441, predicted molecular weight: 42 kDa) expression levels that were used for normalization (b). Experiment was performed 3 times in duplicates or triplicates and data represent mean +/- sd. * $P < 0.05$.

Suppl. Table 1: Top ten upregulated genes in co-cultured endothelial cells.

Gene	Gene description	log2 FC (co- vs. mono-culture)	FDR p-value
ANKRD1	Ankyrin repeat domain 1 (cardiac muscle)	2.5	1.05E-05
IL1RL1	Interleukin 1 receptor-like 1	2.3	5.29E-07
SULF1	Sulfatase 1	2.1	0.0001
PALMD	Palmdelphin	1.8	0.0009
CFH	Complement factor H	1.8	0.0008
SERPINA1	Serpin peptidase inhibitor, clade A (alpha-1 antiproteinase, antitrypsin), member 3	1.6	0.0252
C1R	Complement component 1, r subcomponent	1.6	0.0388
BMP4	Bone morphogenetic protein 4	1.5	0.0048
CHI3L1	Chitinase 3-like 1 (cartilage glycoprotein-39)	1.5	0.0076
BGN	Biglycan	1.5	0.0348

Top ten upregulated genes in co-cultured endothelial cells. Log2 Fold Changes (log2FC) and adjusted P-values are depicted in the third and fourth column, respectively.

Suppl. Table 2: Top ten downregulated genes in co-cultured endothelial cells.

Gene	Gene description	log2 FC (co- vs. mono-culture)	FDR p-value
CXCL8	Chemokine (C-X-C motif) ligand 8	-3.7	1.68E-07
FABP3	Fatty acid binding protein 3, muscle and heart	-3.5	1.28E-08
G0S2	G0/G1 switch 2	-3.4	1.31E-07
TNFSF18	Tumor necrosis factor (ligand) superfamily, member18	-3.1	1.68E-07
ASNS	Asparagine synthetase (glutamine-hydrolizing)	-3.0	1.68E-07
PSAT1	Phosphoserine aminotransferase 1	-2.9	9.96E-7
STC2	Stanniocalcin 2	-2.7	3.49E-05
TFPI2	Tissue factor pathway inhibitor 2	-2.5	1.68E-07
BIRC3	Baculoviral IAP repeat containing 3	-2.4	5.43E-05
SNAI2	Snail family zinc finger 2	-2.4	0.0408

Top ten downregulated genes in co-cultured endothelial cells. Log2 Fold Changes (log2FC) and adjusted P-values are depicted in the third and fourth column, respectively.
